# Pharmacological non-hormonal treatment options for male infertility: a systematic review and network meta-analysis

**DOI:** 10.1186/s12894-024-01545-1

**Published:** 2024-07-29

**Authors:** Bassel H. Al Wattar, Michael P. Rimmer, Jhia J. Teh, Scott C. Mackenzie, Omar F. Ammar, Carolyn Croucher, Eleni Anastasiadis, Patrick Gordon, Allan Pacey, Kevin McEleny, Phillipa Sangster

**Affiliations:** 1 Beginning Assisted Conception Unit, Epsom and St Helier University Hospitals, London, UK; 2 Comprehensive Clinical Trials Unit, Institute of Clinical Trials & Methodology, University College London, London, UK; 3 Clinical Trials Unit, Faculty of Health, Medicine and Social Care, Anglia Ruskin University, Chelmsford, UK; 4 Centre for Reproductive Health, Institute of Regeneration and Repair, University of Edinburgh, Edinburgh, UK; 5 Department of Metabolism, Digestion and Reproduction, Imperial College London, London, UK; 6 Ar-Razzi Hospital, Ramadi, Iraq; 7 Department of Urology, Croydon Health Services NHS Trust, London, UK; 8 Department of Urology, Leeds Teaching Hospitals NHS Trust, Leeds, UK; 9 School of Medical Sciences, Faculty of Biology, Medicine and Health, The University of Manchester, Manchester, UK; 10 Newcastle Fertility Centre at LIFE, Newcastle-upon-Tyne Hospitals, Newcastle upon Tyne, UK; 11 Reproductive Medicine Unit, Elizabeth Garrett Anderson Wing, University College London Hospitals NHS Trust, London, UK

**Keywords:** Male infertility, Oligospermia, Clomiphene, Tamoxifen, Letrozole, Anastrozole, Randomised trials, Systematic review, Network meta-analysis

## Abstract

**Background:**

Male factor infertility affect up to 50% of couples unable to conceive spontaneously. Several non-hormonal pharmacological treatments have been proposed to boost spermatogenesis and increase chances of conception in men with infertility. Still, no clear evidence exists on the most effective treatment strategy.

**Objective:**

We aimed to compare the effectiveness of non-hormonal pharmacological treatment options for men with infertility using a systematic review and network meta-analysis.

**Methods:**

We searched MEDLINE, EMBASE, and CENTRAL until October 2023 for randomised/quasi-randomised trials that evaluated any non-hormonal pharmacological treatment options for men with idiopathic semen abnormalities or those with hypogonadism. We performed pairwise and network meta-analyses using a random effect model. We assessed risk of bias, heterogeneity, and network inconsistency. We calculated the mean rank and the surface under the cumulative ranking curve (SUCRA) for each intervention the maximum likelihood to achieve each of reported outcomes. We reported primarily on sperm concentration and other important semen and biochemical outcomes using standardised mean difference (SMD) and 95% confidence-intervals(CI).

**Results:**

We included 14 randomised trials evaluating four treatments (Clomiphene citrate, Tamoxifen, Aromatase inhibitors, anti-oxidants) and their combinations in 1342 men. The overall quality of included trials was low. Sperm concentration improved with clomiphene compared to anti-oxidants (SMD 2.15, 95%CI 0.78–3.52), aromatase inhibitor (SMD 2.93, 95%CI 1.23–4.62), tamoxifen (SMD − 1.96, 95%CI -3.57; -0.36) but not compared to placebo (SMD − 1.53, 95%CI -3.52- 0.47). Clomiphene had the highest likelihood to achieve the maximum change in sperm concentration (SUCRA 97.4). All treatments showed similar effect for sperm motility, semen volume, and normal sperm morphology. FSH levels showed significant improvement with clomiphene vs.anti-oxidant (SMD 1.48, 95%CI 0.44–2.51) but not compared to placebo. The evidence networks for LH and testosterone suffered from significant inconsistency (*p* = 0.01) with similar trend of improvement with clomiphene compared to other treatments but not compared to placebo.

**Conclusion:**

There is insufficient evidence to support the routine use of Clomiphene, tamoxifen, and aromatase inhibitors to optimise semen parameters in men with infertility. Future randomised trials are needed to confirm the efficacy of clomiphene in improving fertility outcomes in men.

**PROSPERO:**

CRD42023430179.

**Supplementary Information:**

The online version contains supplementary material available at 10.1186/s12894-024-01545-1.

## Background

Infertility is a common disease affecting 8–12% of the world’s population with 1 in 7 heterosexual couples seeking fertility treatments [1]. Male factor infertility defined by abnormal semen parameters affects up to 50% of couples (either as sole factor in 20% or joint male/female in 30% of cases) [2]. The commonest presentation for male infertility is with a combination of reduced sperm concentration, poor sperm motility, and abnormal sperm morphology all of which correlate with reduced ability for natural conception [3]. Male infertility and abnormal spermatogenesis have been attributed to several causes including genetic disorders, environmental factors, chronic illness and neoplasm. Still, more than half of all cases are considered idiopathic or are attributed to subclinical hypogonadism [4]. While common, effective medical treatments for men with hypogonadism or idiopathic infertility remains limited with an over reliance on the use of expensive assisted reproductive technology.

Inadequate stimulation of the testes through the hypothalamic-pituitary-gonadal (HPG) axis commonly results in impaired testicular function and abnormal spermatogenesis [5]. Interventions aimed at boosting testicular function could help to resolve male infertility, increase the chances of natural conception, and reduce the need for assisted conception [6].

Several pharmacological hormonal and non-hormonal interventions have been proposed to boost spermatogenesis. Specifically, selective estrogen receptor modulators (SERMs) (e.g. Clomiphene citrate and Tamoxifen) and aromatase inhibitors (AIs) (e.g. letrozole and anastrozole) are commonly used as off-license treatments for men with idiopathic infertility [7]. Several interventional and observational studies suggested an improvement by using these non-hormonal treatments to increase sperm concentration and circulating Testosterone [8, 9]. However, current evidence remains limited to pair-wise comparisons across studies with varied methodological limitations.

We aimed to leverage both direct and mixed evidence to evaluate the effectiveness of these non-hormonal pharmacological treatment options compared to placebo and to each other in a systematic review and network meta-analysis.

## Materials and methods

We conducted this review following a prospectively registered protocol (CRD42023430179) and reported the findings as per established guidelines [10].

### Eligibility criteria, information sources, search strategy

We searched the following electronic databases (MEDLINE, EMBASE, and Cochrane CENTRAL) for randomised trials evaluating any treatment option for tubal ectopic pregnancy (from inception till October 2023). We used MeSh headings and keywords combined using Boolean operators (AND, OR) to conduct our searches and adjusted the strategy for each database (Supplementary Appendix 1). We conducted supplementary searches in Google Scholar and Scopus to identify any missing evidence. No search filters or language limitations were employed, articles in non-English language were obtained and translated if deemed relevant. We manually screened bibliographies of potentially relevant articles and published systematic reviews on the topic to identify any additional relevant trials. We did not include any unpublished data in the analysis or studies made available online that were not peer-reviewed.

### Study selection

We performed the study selection and data extraction processes in duplicate by three reviewers (JJT, SCM, and OFA) and double checked by two independent reviewers (MPR and BHA). Inconsistencies and disagreement were resolved by discussion and consensus. We included all randomised trials that evaluated the effectiveness of any non-hormonal pharmacological treatment options for more than 3 months in men diagnosed with abnormal semen parameters as per the WHO criteria [11]. We included studies with multiple comparison arms, and those evaluating a combination of treatment options (e.g. clomiphene citrate + anti-oxidants). For this review, we considered the use of placebo and no active treatment to be equal. We also considered the use of any herbal or active medical treatment option aimed at reducing oxidative stress and improving sperm quality as an anti-oxidant (Supplementary Table 1). We excluded studies that evaluated a hormonal treatment (e.g. exogenous testosterone) alone or in combination with a non-hormonal treatment option (e.g. tamoxifen). We also excluded studies of non-randomised design, cross over trials, those not reporting on any semen parameters or biochemical outcomes post treatment, those that evaluated the intervention after less than 3 months of active treatment, and studies in animals.

### Data extraction

Three independent reviewers extracted data in duplicate (JJT, SCM, and OFA). We extracted data onto a bespoke data extraction tool that was piloted prospectively among the reviewers for validity and reliability. Two independent reviewers (MR and BHA) checked the quality and integrity of 10% of the data and resolved any inconsistencies. Our main outcome was the concentration of sperm after a minimum of three months of active treatment. We also reported on other semen parameters (total sperm count, total motility percentage, total percentage of normal sperm morphology), biochemical outcomes (FSH, LH, testosterone, estradiol) and reproductive outcomes (clinical pregnancy, miscarriage, stillbirth, live birth).

We extracted data on the country of the study, the publication journal, trial settings, population characteristics, the characteristic and duration of treatment, the length of follow-up and the causes for infertility.

### Assessment of risk of bias

Three independent reviewers (JJT, SCM, and OFA) assessed the quality of included studies using the Cochrane risk of bias assessment tool 2 [12]. Each study was assessed for the quality of randomisation and sequence generation, allocation to intervention groups, outcome assessment, completeness of outcome data, and selective outcome reporting. Efforts to standardise the semen analysis and other outcome assessment measures were considered to show less risk of bias when evaluating detection and performance bias.

### Data synthesis

First, we assessed the network geometry of available evidence comparing all available treatment options using the network map command in Stata. Where possible, we generated pool effect estimates from direct comparisons of each treatment pair using a random-effect REML model. We assessed heterogeneity using I^2^ statistics and explored potentials for risk of publication bias using a funnel plot. We then performed a network meta-analysis within a frequentist framework fitting multivariate meta-analysis models with random effect using the network package in Stata [13, 14] exploiting the direct and indirect randomised evidence to determine the relative effects and ranking. We reported the effects of the interventions using standardised mean difference (SMD) for continuous outcomes and risk ratios (RR) for dichotomous outcome with 95% confidence intervals (CI). We adopted a pragmatic approach whereby all pharmacological agents were considered equal irrespective of dose, duration, and frequency of use. Where a trial reported outcome at several intervals, we included data from the longest follow up duration (6 over 3 months follow up). We calculated the mean rank and the surface under the cumulative ranking curve (SUCRA) for each intervention the maximum likelihood to achieve each of reported outcomes. Treatment options with a SUCRA value close to 100% had the highest cumulative rank (i.e. highest likelihood) for achieving the reported outcome [15, 16].

We used the design-by-treatment model to check the assumption of consistency in the entire network assuming a common estimate for the heterogeneity variance across the different comparisons in the network for each of the reported outcomes [17]. Where relevant, we investigated and detected inconsistency by comparing the direct and indirect evidence within the network using the node-splitting approach assuming a common heterogeneity estimate within each loop [18], as well as investigating potential sources of inconsistency within relevant trials. We planned a network meta-regression to explore any detected inconsistency for potential effect modifiers [19]. All analyses were done using Stata statistical software, release 18.0 (StataCorp, College Station, TX).

## Results

### Study selection and study characteristics

**Fig. 1:**
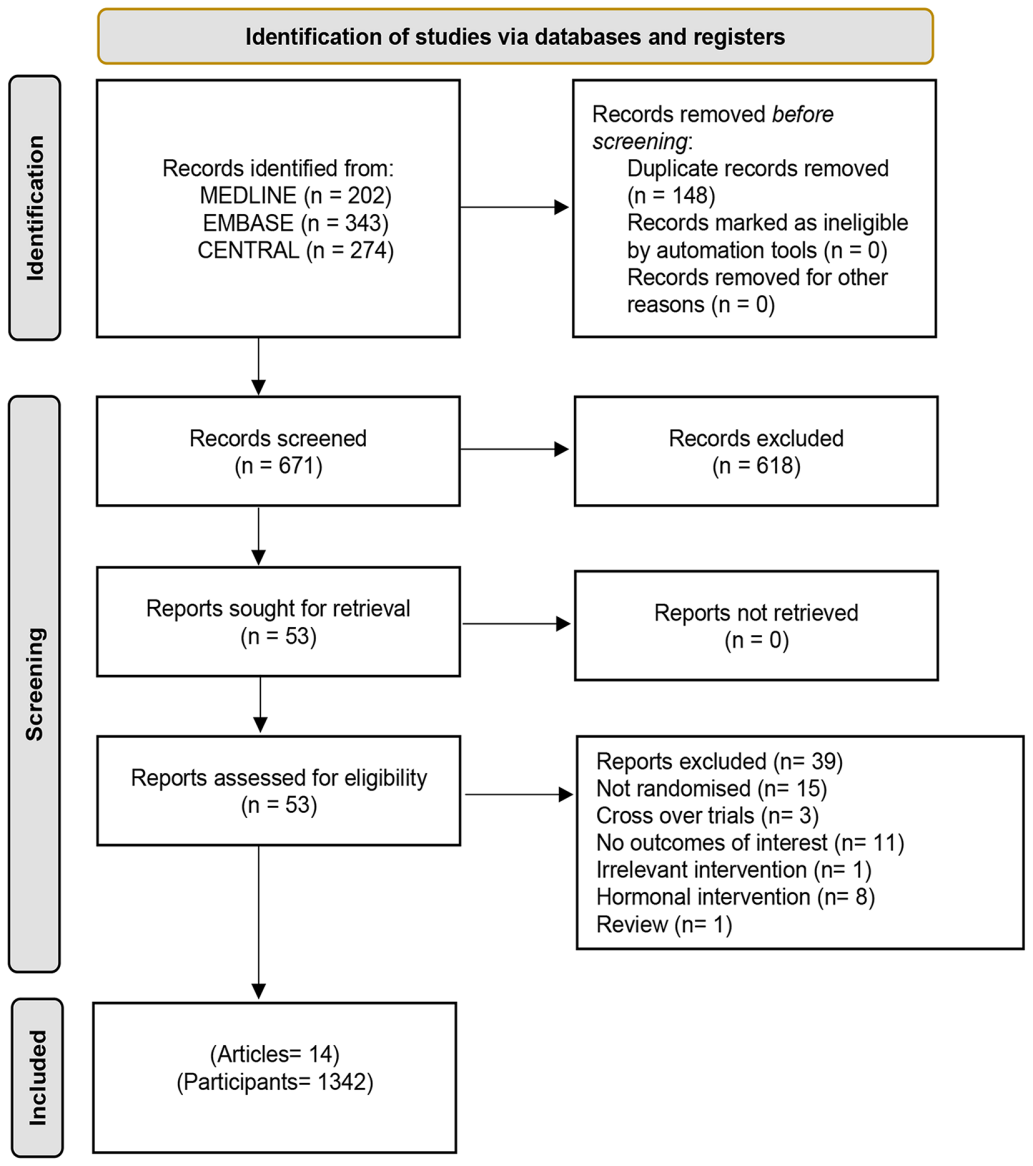
Study selection and inclusion process of randomised trials evaluating non-hormonal pharmacological treatment options for male infertility

Our electronic search yielded 671 potentially relevant citations out of which we screened 53 in full and included 14 randomised/quasi-randomised trials in our review (Fig. 1). Ten of the included trials had a two groups parallel design, three had three groups [20, 21]and one compared four groups [22]. Majority of the trials were single centre (11/14, 78.6%) and only three were multi-centre (3/14, 21.4%). Most of the trials were conducted in Asia (6/14, 42.9%) followed by four in Europe (4/14, 28.6%), and two in each of the USA and Egypt (2/14, 14.3%).

The majority of the trials included men with low sperm count (< 15 × 10^6/ml) with only two trials including men with very low sperm count (< 5 × 10^6/ml) [21, 23](Supplementary Table 1). Only three trials included men with hypogonadism [21, 23, 24] and one trial provided the intervention for 12 months [25] with eleven reporting outcomes between three and six months from recruitment (11/14, 78.6%).

We compared four pharmacological treatment options in our network meta-analysis (*n* = 1342) including Clomiphene citrate; Tamoxifen; Aromatase inhibitors (1 RCT for anastrazole, 2 RCTs for letrozole); anti-oxidants; and their combinations (Clomifene + Anti-oxidant; Tamoxifen + Anti-oxidant) (Supplementary Table 1).

### Risk of bias of included studies

The overall quality of included trials was low. Half of the included trials (7/14, 50%) showed high risk of bias for randomisation and half showed some concern for allocation to intervention groups (7/14, 50%). Two trials were published as randomised trials, but the reported methods of randomisation were more consistent with quasi-randomisation [20, 26]. Seven trials showed high risk for outcome assessment (7/14, 50%) and eleven selective reporting bias (11/14, 79%). Two trials (2/14, 14%) had high loss to follow-up and data incompleteness (Supplementary Fig. 1, Supplementary Table 2). We assessed the risk of publication bias in included trials visually using a funnel plot which suggested no significant small study effect (Supplementary Fig. 3).

### Synthesis of results

#### Semen parameters

A direct meta-analysis was only possible for clomid vs. placebo from three RCTs [25–27] as all other treatment comparisons came from less than three trials each. Overall, there was no significant improvement in sperm concentration (SMD 0.71 95%CI -0.18 to 1.61, I^2^ 91%) with some improvement in sperm motility (SMD 0.44 95%CI 0.21 to 0.66, I^2^ 0) (Supplementary Fig. 3).

**Fig. 2:**
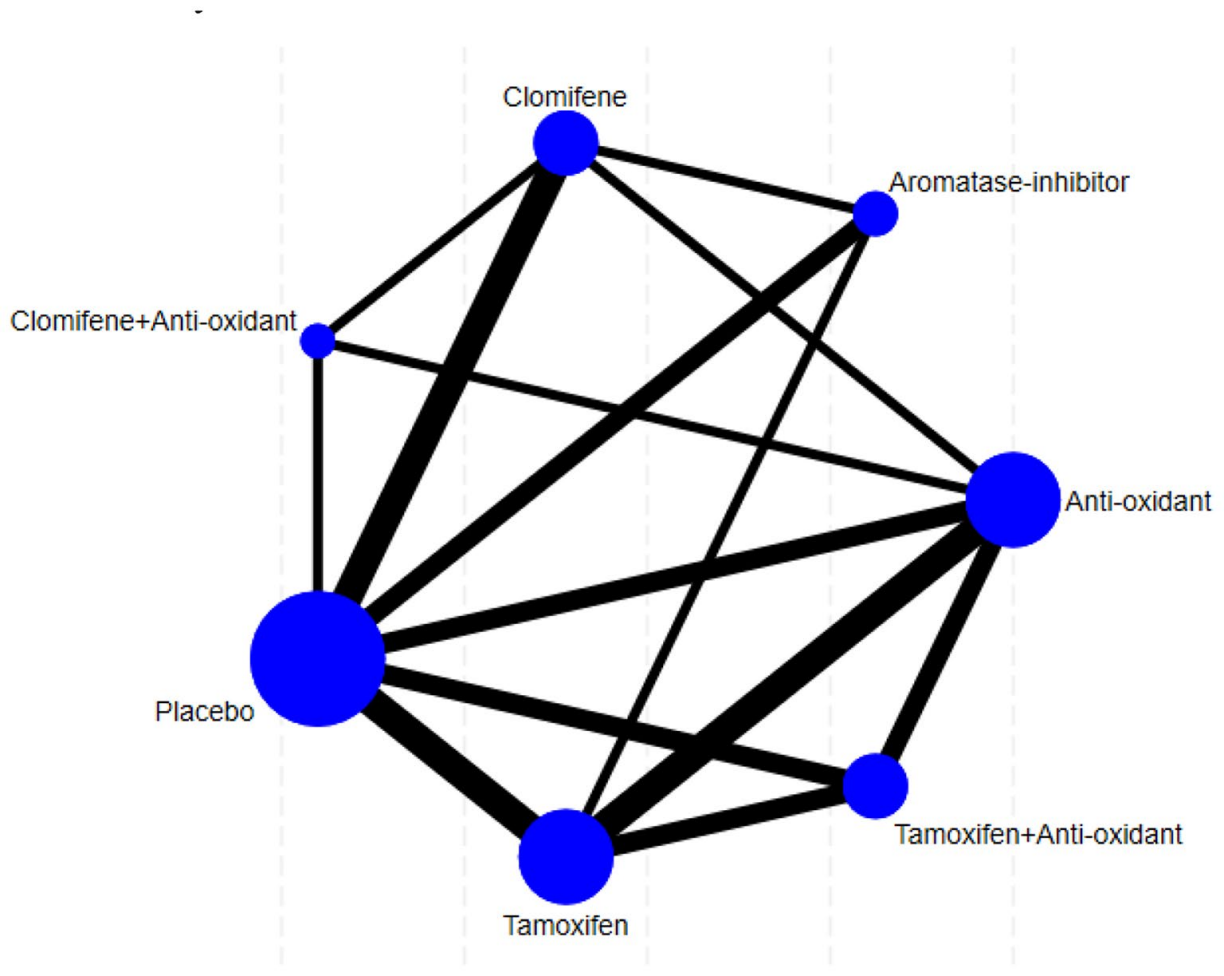
Network of randomised trials comparing non-hormonal pharmacological treatment options for male infertility. The dots’ size represents the number of participants in each comparison arm and the lines’ thickness represent the number of randomised trials comparing each two treatments directly

**Fig. 3:**
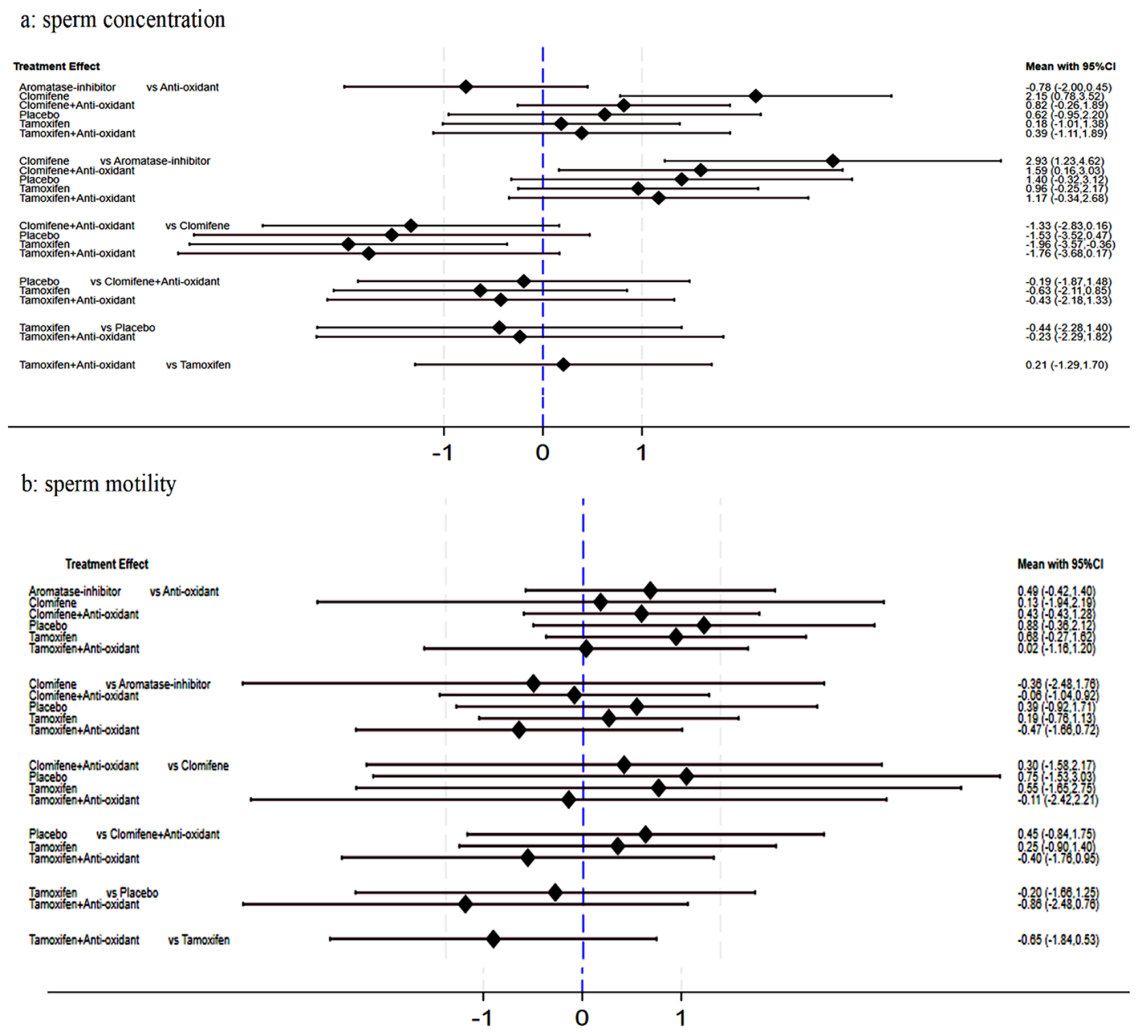
Forest plots of network meta-analysis of changes in sperm concentration and motility across non-hormonal pharmacological treatment options for male infertility

**Fig. 4:**
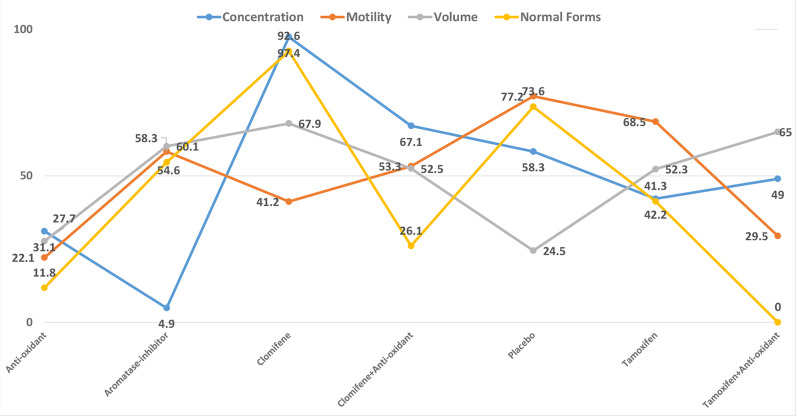
Surface under the cumulative ranking curve of changes in semen parameters following the use of non-hormonal pharmacological treatment options for male infertility. Surface under the cumulative ranking curve expressed in percentage. Higher values suggest higher likelihood of the treatment achieving the outcome of interest

Our network meta-analysis (Fig. 2) showed significant improvement in sperm concentration with clomiphene compared to other treatment options (clomiphene vs. anti-oxidant (SMD 2.15 95%CI 0.78 to 3.52), clomiphene vs. aromatase inhibitor (SMD 2.93 95%CI 1.23 to 4.62), tamoxifen vs. clomiphene (SMD − 1.96 95%CI -3.57 to -0.36) although there was no significant difference for placebo vs. clomiphene (SMD − 1.53 95%CI -3.52 to 0.47) (Fig. 3, Supplementary Fig. 4b). All other treatment comparisons showed no significant difference compared to placebo (Fig. 3, Supplementary Fig. 4b). The network did not suffer from significant inconsistency (*p* = 0.178). Clomiphene had the highest likelihood to achieve the maximum change in sperm concentration (SUCRA 97.4) followed by Clomifene + Anti-oxidant (SUCRA 67.1) (Fig. 4). Aromatase-inhibitor had the lowest likelihood of demonstrating maximum improvement in sperm concentration with a SUCRA of 4.9 (Fig. 4, Supplementary Fig. 4c).

All treatments showed similar effect for sperm motility compared to each other and placebo (Fig. 3, Supplementary Fig. 5) with similar ranking likelihood and no significant network inconsistency (*p* = 0.97) (Fig. 4, Supplementary Fig. 5). Similarly, no significant difference was noted across all treatments for changes in semen volume (Supplementary Fig. 6) with both clomiphene (SUCRA 67.9) and Aromatase-inhibitor (SUCRA 60.1) showing greatest likelihood of achieving an improvement in semen volume (Fig. 4, Supplementary Fig. 6).

Both clomiphene (SMD 3.01 95%CI 0.55 to 5.47) and placebo (SMD 1.77 95%CI 0.30 to 3.25) showed a significant improvement in normal sperm morphology compared to anti-oxidants with no other significant effect for any of the remaining treatment options (Supplementary Fig. 7). However, clomiphene had a higher rank (SUCRA 92.6) compared to placebo (SUCRA 73.6) in achieving a greater improvement in sperm normal morphology. This network did not suffer from significant inconsistency (*p* = 0.76) (Fig. 4, Supplementary Fig. 7).

### Biochemical outcomes

There was limited reporting for other planned secondary outcomes and a direct pairwise meta-analysis was not possible for any of the reported biochemical outcomes.

Network meta-analysis for changes in FSH levels showed significant improvement with clomiphene vs. anti-oxidant (SMD 1.48 95%CI 0.44 to 2.51) but with no significant difference for clomiphene vs. placebo (SMD − 1.13 95%CI -2.40 to 0.15) (Supplementary Fig. 8b). All other treatments showed similar effect with clomiphene (SUCRA 80.6) and tamoxifen (SUCRA 82.7) showing the highest likelihood for an increase in FSH. There was no significant inconsistency in this network (*p* = 0.73) (Supplementary Fig. 8c).

The network for evaluating the impact on LH suffered from significant inconsistency (*p* = 0.01) and we therefore applied an inconsistency model(Supplementary Fig. 9). Overall, aromatase inhibitors significantly increased LH levels compared to all other treatments (aromatase-inhibitors vs. anti-oxidant (SMD 3.20 95%CI 1.94 to 4.45); clomiphene vs. aromatase-inhibitors (SMD − 1.65 95%CI -3.31 to 0.02); placebo vs. aromatase-inhibitors (SMD − 1.58 95%CI -3.08 to -0.08); tamoxifen vs. aromatase-inhibitors (SMD − 1.34 95%CI -2.47 to -0.21); tamoxifen + anti-oxidant vs. aromatase-inhibitors (SMD − 1.26 95%CI -2.39 to -0.13)) (Supplementary Fig. 9b) and had the highest likelihood for improving LH (SUCRA 98.8) (Supplementary Fig. 9c). Similarly, tamoxifen + anti-oxidant showed significant improvements compared to anti-oxidants only (SMD 1.62 95%CI 0.80 to -2.44) and placebo was more effective than anti-oxidants only (SMD 1.55 95%CI 0.46 to -2.64) (Supplementary Fig. 9b).

The network for evaluating the impact on Testosterone suffered from significant inconsistency (*p* = 0.01) and we therefore employed an inconsistency model(Supplementary Fig. 10). Clomiphene significantly increased Testosterone compared to other treatments (clomiphene vs. anti-oxidants (SMD 2.06 95%CI 1.34 to -2.79); clomiphene vs. aromatase-inhibitors (SMD 2.57 95%CI 1.25 to 3.89); tamoxifen vs. clomiphene (SMD − 1.16 95%CI -2.03 to -0.30) but not compared to placebo (SMD 0.04 95%CI -0.85 to 0.93)). All of the other active treatments did not show significant effect compared to placebo (placebo vs. anti-oxidants (SMD 2.10 95%CI 1.33 to 2.87); placebo vs. aromatase-inhibitors (SMD 2.61 95%CI 1.23 to 3.98); tamoxifen vs. placebo (SMD − 1.20 95%CI -2.18 to -0.22); tamoxifen + anti-oxidants vs. placebo (SMD − 0.60 95%CI -1.99 to 0.79) (Supplementary Fig. 10b). Clomiphene had the highest likelihood of achieving an increase in Testosterone (SUCRA 82.3) followed by Tamoxifen + Anti-oxidant (SUCRA 76.9) (Supplementary Fig. 10c).

We explored sources of inconsistency with a sidesplit approach. Significant inconsistency emerged from trials that include a placebo or anti-oxidants comparison arm (Supplementary Table 3).

### Reproductive outcomes

Only three trials reported on clinical pregnancy [28, 25, 29] and we were unable to capture any of the other planned reproductive outcomes in our protocol. A meta-analysis was not possible although there was a reported increase in pregnancy with the use of clomiphene vs. placebo in two trials [29, 25] and with tamoxifen vs. placebo in one trial [28] (Supplementary Table 4).

## Discussion

### Principal findings

In this network meta-analysis, we evaluated the efficacy of available non-hormonal pharmacological treatment options for men with infertility across a total of fourteen randomised/quasi-randomised trials. While clomiphene seems to outperform other treatment options to achieve an improvement in both semen parameters as well as biochemical outcomes, there was no significant improvement with its use compared to placebo. The use of several anti-oxidants seemed to not yield any significant difference alone or when combined with other active treatments (e.g. clomiphene and tamoxifen). The use of aromatase inhibitors may alter some hormonal function (namely LH), but this did not translate to improvement in semen parameters across included trials. Overall, none of the evaluated treatment options demonstrated efficacy across reported outcomes compared to placebo, and therefore, their clinical value remains uncertain.

### Strengths and limitations

Our review adopted a standard methodology and leveraged both direct and indirect evidence from available trials to synthesise precise effect estimates [30]. We assessed the quality of available trials, the risk of publication bias, and explored sources of inconsistency where relevant.

Our results suffered from several limitations. Firstly, the quality of included trials was low with a significant risk of bias across different domains especially due to poor randomisation and outcome reporting methodology (Supplementary Fig. 1). This likely to have contributed to the perceived heterogeneity and inconsistency in evidence networks on several key outcomes (LH and testosterone). We explored sources of inconsistency using a side-split approach which seems to stem from trials with placebo arms. Several factors could have contributed to this including variations in investigational medicinal product, blinding procedures, and lab assays across included trials. We employed both consistency and inconsistency random effect-models to adjust for the perceived network inconsistency and explored sources of inconsistency where possible. Due to limited number of trials involving placebo, a rule-one-out analysis was not feasible.

We planned to conduct subgroup analyses and meta-regression to explore potential confounders including participants age group, BMI, and testosterone/oestrogen ratio. However, this was not possible due to limited reporting across included trials. We opted to a pragmatic approach and combine treatments with similar mechanistic effects to produce higher quality evidence that is relevant to everyday clinical practice.

There was wide variation in outcome reporting which limited our ability to assess all planned outcomes in our protocol such as live birth and use of assisted conception treatments. Our review included trials spanning across four decades (1982 to 2020) during which the methods to evaluate changes in semen parameters have evolved significantly. As such, a degree of outcome assessment bias cannot be ruled out which may limit the applicability of older trials into today’s clinical practice.

### Implications for clinical practice

Empirical pharmacological treatment for men with hypogonadism and/or idiopathic infertility have featured in the medical literature for more than five decades, however, evidence on effective and safe treatment strategy remains inconclusive [31]. In contrast to previously published pairwise meta-analysis of observational and randomised evidence [8, 9], our network meta-analysis showed limited efficacy for all evaluated treatment options over placebo. The decision to start such treatments depends on the underpinning cause of infertility, patient characteristics, and overall treatment objectives.

Most of the included trials evaluated these treatments in men with idiopathic infertility. However, it is important to distinguish the perceived benefit among men with hypogonadism compared to those with normal testosterone levels [32]. Specifically, men with a reduced testosterone/estradiol ratio may see more benefit associated with clomiphene and aromatase inhibitor therapy [32]. Similarly, men with high BMI [33] or advanced age [34] may also demonstrate more benefit following SERMs/AIs therapy compared to those with no other predisposing factors.

The magnitude of benefit may also vary depending on the degree of semen abnormality [31]. While most of the included trial in our review demonstrated some improvement in sperm concentration, the magnitude of changes was smaller among men with very low sperm count [23]. Due to limited reporting, were unable to further explore the mechanistic effect of evaluated treatment on varied semen abnormalities (e.g. low vs. very low sperm count).

The desired benefit should, therefore, be considered within the overall treatment strategy. Adopting non-hormonal pharmacological treatments could help to boost the chances of natural conception in couple with mild male factor infertility [26]. It could also be adopted as intermediate treatment to increase the chances of surgically retrieving good quality sperm in men with very low sperm count and maximise the chance of conception with assisted conception [28].

Finally, most of the included trials introduced the treatment for a short period of time (3–6 months) with limited reporting on longterm clinical and reproductive outcomes. Both SERMS and AI are well tolerated with limited profile of side effects [7], however, careful monitoring and surveillance is recommended for prolonged use beyond what is reported in our meta-analysis.

### Future research need

Clomiphene consistently ranked as the most likely treatment to improve clinical outcomes in men with infertility over other treatment options, however, it did not demonstrate significant efficacy compared to placebo. Due to suboptimal trial methodology, none of the included trials reported sufficient longitudinal outcomes to enable adequate evaluation of the mechanistic effect of clomiphene in the trial cohort compared to placebo. There is a need for an adequately powered randomised trial to confirm the true efficacy of clomiphene on the semen parameters, biochemical, hormonal, and clinical outcomes of men with infertility. Specifically, there is a need for an extended evaluation of the longterm reproductive outcomes with the use of clomiphene, especially regarding its safety and tolerability.

There is a need to explore the effect of potential effect modifiers (e.g. age, BMI, dose, and adherence). Given the paucity of trials and limited reporting, prospective individual participant meta-analysis of future trial is required to achieve this objective. Future IPD meta-analyses using individual data from both randomised and observational studies with adequate adjustment for key confounders could also offer higher quality evidence and inform the design of future randomised trials.

None of the included trials sought input from lay consumers on study design or the choice of outcome reporting. Beyond conception, these pharmacological treatments could have a significant impact on men’s quality of life which was reported only in one trial [24]. Incorporating patient reported outcomes measures is critical in future trials as well as adopting established core outcome sets [35].

## Conclusions

There is insufficient evidence to support the routine use of Clomiphene, tamoxifen, and aromatase inhibitors to optimise semen parameters in men with infertility. Future randomised trials are needed to confirm the efficacy of clomiphene in improving fertility outcomes in men.

## Electronic supplementary material

Below is the link to the electronic supplementary material.

Supplementary Material 1

Supplementary Material 2

## Data Availability

Data are available for bona fide researchers who request it from the authors.
