# Correction: Pharmacological non-hormonal treatment options for male infertility: a systematic review and network meta-analysis

**DOI:** 10.1186/s12894-025-01874-9

**Published:** 2025-07-18

**Authors:** Bassel H. Al Wattar, Michael P. Rimmer, Jhia J. Teh, Scott C. Mackenzie, Omar F. Ammar, Carolyn Croucher, Eleni Anastasiadis, Patrick Gordon, Allan Pacey, Kevin McEleny, Phillipa Sangster

**Affiliations:** 1 Beginning Assisted Conception Unit, Epsom and St Helier University Hospitals, London, UK; 2 Comprehensive Clinical Trials Unit, Institute of Clinical Trials & Methodology, University College London, London, UK; 3 Clinical Trials Unit, Faculty of Health, Medicine and Social Care, Anglia Ruskin University, Chelmsford, UK; 4 Centre for Reproductive Health, Institute of Regeneration and Repair, University of Edinburgh, Edinburgh, UK; 5 Department of Metabolism, Digestion and Reproduction, Imperial College London, London, UK; 6 Ar-Razzi Hospital, Ramadi, Iraq; 7 Department of Urology, Croydon Health Services NHS Trust, London, UK; 8 Department of Urology, Leeds Teaching Hospitals NHS Trust, Leeds, UK; 9 School of Medical Sciences, Faculty of Biology, Medicine and Health, The University of Manchester, Manchester, UK; 10 Newcastle Fertility Centre at LIFE, Newcastle-upon-Tyne Hospitals, Newcastle upon Tyne, UK; 11 Reproductive Medicine Unit, Elizabeth Garrett Anderson Wing, University College London Hospitals NHS Trust, London, UK

**Correction: BMC Urol 24, 158 (2024)**

**https://doi.org/10.1186/s12894-024-01545-1**

In this article [1], the author’s name Jhia J. Teh was incorrectly written as Jack J. Teh.

The original article has been corrected.
