# Supplementary material for: Pharmacological non-hormonal treatment options for male infertility: a systematic review and network meta-analysis
Source: BMC Urol. 2024 Jul 29;24:158. doi: 10.1186/s12894-024-01545-1 (PMC11285325; doi:10.1186/s12894-024-01545-1)

**Supplementary materials**

**Pharmacological treatment options for male infertility: a systematic review and network meta-analysis.**

Bassel H.Al Wattar^1,2,3^, Michael P Rimmer^4^, Jack J Teh^5^, Scott C Mackenzie^6^, Omar F Ammar^7^, Carolyn Croucher^1^, Eleni Anastasiadis^8^, Patrick Gordon^9^, Allan Pacey^10^, Kevin McEleny^11^, Phillipa Sangster^12^

- **List of abbreviations**
- **Supplementary Appendix (1):** Literature search strategy for randomised trials evaluating non-hormonal pharmacological treatment options for male infertility.
- **Supplementary Table (1):** Characteristics of included randomised trials evaluating non-hormonal pharmacological treatment options for male infertility.
- **Supplementary Table (2):** Risk of bias across individual randomised trials included in the network meta-analysis evaluating non-hormonal pharmacological treatment options for male infertility.
- **Supplementary Table (3):** Side-split analysis of inconsistency in the network for testosterone in the network meta-analysis evaluating non-hormonal pharmacological treatment options for male infertility.
- **Supplementary Table (4):** Summary of reported reproductive outcomes across included trials evaluating non-hormonal pharmacological treatment options for male infertility.
- **Supplementary Figure (1):** Summary of risk of bias across included randomised trials evaluating non-hormonal pharmacological treatment options for male infertility.
- **Supplementary Figure (2):** Funnel plot evaluating the risk of publication bias in included randomised trials evaluating non-hormonal pharmacological treatment options for male infertility.
- **Supplementary Figure (3):** Direct meta-analysis of changes in sperm concentration and motility with the use of Clomiphene vs placebo in men with infertility.
- **Supplementary Figure (4):** Network meta-analysis of changes in sperm concentration across non-hormonal pharmacological treatment options for male infertility.
- **Supplementary Figure (5):** Network meta-analysis of changes in sperm motility across non-hormonal pharmacological treatment options for male infertility.
- **Supplementary Figure (6):** Network meta-analysis of changes in semen volume across non-hormonal pharmacological treatment options for male infertility.
- **Supplementary Figure (7):** Network meta-analysis of changes in normal sperm morphology across non-hormonal pharmacological treatment options for male infertility.
- **Supplementary Figure (8):** Network meta-analysis of changes in FSH across non-hormonal pharmacological treatment options for male infertility.
- **Supplementary Figure (9):** Network meta-analysis of changes in LH across non-hormonal pharmacological treatment options for male infertility.
- **Supplementary Figure (10):** Network meta-analysis of changes in testosterone across non-hormonal pharmacological treatment options for male infertility.

**List of abbreviations:**

SUCRA: Surface under the cumulative ranking curve

NR: Not Reported

COI: conflict of interest

**Supplementary Appendix (1):** Literature search strategy for randomised trials evaluating non-hormonal pharmacological treatment options for male infertility.

-clomid OR clomiphene OR tamoxifen OR letrozole OR anastrozole OR testolactone OR Aromatase Inhibitors OR Selective estrogen receptor modulators OR SERM

-men OR man OR male

-infertility OR subfertility OR infertile OR conception OR pregnancy

-semen OR sperm OR spermatozoa OR spermatid

-randomized OR randomised

**Supplementary Table (1):** Characteristics of included randomised trials evaluating non-hormonal pharmacological treatment options for male infertility.

| Study | Country | N | Setting | Inclusion criteria | Exclusion criteria | Diagnostic criteria for male subfertility | Intervention | Comparison | Treatment duration | Follow up duration |
| --- | --- | --- | --- | --- | --- | --- | --- | --- | --- | --- |
| Abel 1982 | UK | 196 | Multicentre | Men with infertility with no obvious endocrinopathy. | Men with azoospermia, varicocele or undescended testis and men with partners with bilateral fallopian tube obstruction. | Not stated | Clomiphene 50 mg/day | Vitamin C 200 mg/day | 6 months | 3 months |
| Boonyarangkul 2015 | Thailand | 68 | Single centre | Abnormal semen analysis of at least one parameter according to World Health Organization Criteria 2010 (concentration <15x10^6/ml, motility <40%, or morphology <4%), failure of the female partner to conceive after one year of regular unprotected sexual intercourse, no history of Tamoxifen and Folate allergy | Use of tamoxifen and folate within three months before recruitment, use of other medicines or vitamin during study period | Abnormal semen analysis of at least one parameter according to WHO 2010 (concentration <15X10^6/ml, motility <40%, or morphology <4%) | Tamoxifen 20 mg/day | Placebo | 3 months | 3 months and 6 months |
| Cavallini 2013 | Italy | 52 | Multicentre | Non-smoker, non-obstructive azoospermia patient on FNA and cryptozoospermic patients with T/E2 ratio <10. normal sperm appearance, consistency, liquefaction, volume, and pH and no chromosomal aberrations. | Seminal white blood cell concentration >106/ml and/or a positive seminal cultural analysis or positive urethral swab chlamydia test; drug, tobacco, or alcohol abuse; ongoing medical treatment (gonadotropins, anabolic steroids, cancer chemo- therapy, non-steroidal anti-inflammatory drugs; previous cancer radiotherapy or chemotherapy, palpable varicocele; X-ray exposure in the previous 8 months; Y chromosome microdeletion, and karyotype alterations) | Azoospermia was defined as the absence of sperm in the pellets of two centrifuged (300g for 15 min) semen samples collected 7–30 days apart; crypto- azoospermia was defined as the presence of sperm in the pellet (but not in the ejaculate) of at least one semen sample out of the two collected | Letrozole 2.5mg/day | Placebo | 6 months | 3 months and 6 months |
| El-Sheikh 2015 | Egypt | 90 | Single centre | 1 year of primary infertility with persistent idiopathic oligoasthenozoospermia | Smokers, heavy smokers with < 6/12 abstinence and systemic illness, endocrine disease, genital tract infection, recurrent or residual varicocele, and history of cryptorchidism, orchitis, post-pubertal mumps, or antisperm antibodies or other known etiology of male infertility. | Sperm concentration < 15 million per ml and sperm total motility <40%, with normal sperm morphology | Clomiphene 25 mg/day | Vitamin E 400mg/day, clomiphene 25mg/day and Vitamin E 400mg/day | 6 months | 3 months and 6 months |
| Ghanem 2010 | Egypt | 60 | Single centre | Men aged 20-40 years old >1 year of infertility with 1 year of regular unprotected intercourse. Unexplained repeated oligoasthenozoospermia. Sperm concentration >20 million/ml, sperm total motility >50%, sperm forward progressive motility <25%, normal sperm morphology greater >30%. | Cases with known aetiology or apparent physical finding, leukocytospermia, diminished testicular volume (a minimum of 20 mL as depicted by ultrasonography), varicocele (as detected by clinical examination and ultrasonography) or an abnormal FSH level. Couples with combined male and female factors were excluded. | Sperm concentration >20 million/ml, sperm total motility >50%, sperm forward progressive motility <25%, normal sperm morphology greater >30%. | Clomiphene 25mg/day, Vitamin E 400mg/day | Placebo | 6 months | 0 beyond treatment period |
| Guo 2015 | China | 120 | Single centre | Oligoasthenozoospermia <15 × 10^6/ml or total number of sperm <39 × 10^6, the percentage of forward motile sperm <32 % and unexplained infertility (unsuccessful pregnancy with active and unprotected intercourse for a period of more than 12 months). | Known aetiology or apparent physical defects, leukocytospermia, diminished testicular volume (a minimum of 12ml as depicted by ultrasonography), or abnormal FSH level, severe oligospermia (sperm count <5x10^6/ml), history of epididymis-orchitis, prostatitis, genital trauma, testicular torsion, inguinal or genital surgery, urinary tract infection or previous hormonal therapy, another genital disease (cryptorchidism or varicocele), Y chromosome microdeletions or karyotype abnormalities, occupational and environmental exposure to potential reproductive toxins, combined male and female factors, and the records for use of cancer chemotherapy, testosterone, anti-androgens or antioxidants, and tobacco. | <15 × 10^6/ml or total number of sperm <39 × 10^6, the percentage of forward motile sperm <32 % | Indomethacin 50 mg/day | Tamoxifen 20 mg/day | 3 months | 0 beyond treatment period |
| Haje and Naoom 2015 | Iran | 128 | Single centre | Couples with male cause of infertility with idiopathic oligoazoospermia | Leukocytospermia, altered testicular volume of a minimum of 20 ml as depicted by ultrasonography,10 varicocele as detected by clinical examination and ultrasonography, abnormal FSH levels, and/or couples with combined male and female factors | Oligoazoospermia defined as per WHO | Tamoxifen 20 mg/day, L-carnitine 1g/day, Tamoxifen 20 mg/day and L-carnitine 1g/day | Placebo | 3 to 6 months | 0 beyond treatment period |
| Helo 2015 | USA | 26 | Single centre | Men aged between 18-50 years old with male infertility and hypogonadism, defined as serum T less than 350 ng/dL and LH between 1.2 and 8.6 mIU/mL | Sperm count <1x10^6, BMI >40, HCT<36% or >52%, history of prostate specific antigen greater than 4.0 ng/dL, history of chronic opioid use, intravenous or inhaled steroid use within the previous 3 months, or use of drugs known to affect steroid hormone or SHBG levels. Additional exclusion criteria included known testicular or pituitary disease, history of prostate cancer or severe benign prostatic hypertrophy. | Idiopathic hypoandrogenism | Clomiphene 25 mg/day | Anastrozole 1 mg/day | 3months | 3 months |
| Krause 1992 | Germany | 76 | Two centres | Sperm count 2-20x10^6/ml; sperm motility = 20-50%; sperm morphology = 50-80% abnormal sperm | Varicocele, history of testicular maldescent, history of genital inflammation and severe general diseases. | Sperm count 2-20x10^6/ml; sperm motility = 20-50%; sperm morphology = 50-80% abnormal sperm | Tamoxifen 20 mg/day | Placebo | 3 months | 3 months |
| Maier 1990 | Austria | 40 | Single centre | Mild oligoasthenozoospermia | Varicocele and/or chronic adnexal infection or hypo­ gonadism | Sperm density of 10-19x10^6/ml and a 2-hour motility between 20-49 % | Tamoxifen 30 mg/day | Tamoxifen 30 mg/day and Testolactone 150 mg/day | 3 months | 0 beyond treatment period |
| Mandal 2020 | India | 200 | Single centre | Healthy males ages 20-45 years old with idiopathic oligospermia, married for >1 year, with no issue giving informed written consent who were willing for treatment for 3 months with monthly follow-up and semen examination | Renal, hepatic or other chronic illness, azoospermia, obesity, male reproductive accessory gland infections, any recent medical or surgical illness, hypertension, oligospermia of definitive pathology, ejaculatory or erectile dysfunctions | Idiopathic oligospermic men (sperm count < 15 million/ml of ejaculate ) | Clomiphene 25 mg/day | Placebo | 3 months | 0 months beyond treatment period |
| Mičič 1985 | Yugoslavia | 101 | Single centre | Idiopathic oligospermia and infertile marriages for more than 2 years, pre-treatment sperm count ranged from 1 to 19 X 10^6/ml, All men had low or normal levels of serum FSH | N/A | Pre-treatment sperm count ranged from 1 to 19 X 10^6/ml, | Clomiphene 50 mg/day | Routine care | 6-9 months | 0 beyond treatment period |
| Moradi 2010 | Iran | 52 | Single centre | 1 year history of infertility and semen parameter abnormalities, including sperm concentration or count < 20 × 10^6 spermatozoa/mL, motility < 50% | Patients with symptoms of hypogonadism, sperm count <10 million/mL, and/or any hormonal disorder detected by hormonal tests, including T, PRL, LH, and FSH, patients with an inguinal operation, infectious or venereal diseases, testicular atrophy, hypogonadism, varicocele, and disorders of the vas deferens and the epididymis were also excluded | Sperm concentration or count < 20 × 10^6 spermatozoa/mL | Clomiphene 25 g/day | L-carnitine 25 mg/day | 3 months | 0 beyond treatment period |
| Sokol 1988 | USA | 23 | Single centre | Men aged 23-49 years old, with infertility, unable to conceive after 1 year of regular unprotected intercourse, three semen analyses in which sperm count ranged between 0.5 and 20 million sperm/ml, sperm morphology was normal, and sperm motility was> 10%; normal serum values for LH, FSH, T and PRL and a female partner who was determined to be fertile | Major systemic or psychiatric illness | Three semen analyses in which sperm count ranged between 0.5 and 20 million sperm/ml, sperm morphology was normal, and sperm motility was> 10%; normal serum LH, FSH, T and PRL | Clomiphene 25 mg/day | Placebo | 12 months | 0 months beyond treatment period |
| Xiaoxia 2019 | China | 150 | Single centre | Men aged 25 to 40 years old, sperm concentration <5×10^6/ml, T<10.4 nmol/L, T/E2 ratio < 0.1, married cohabitation without contraception for >1 year without a history of successful fertility | Congenital malformation of reproductive organs, sexual dysfunction, prostatitis and other diseases affecting fertility, combination of heart, liver, spleen, lung and kidney serious functional insufficiency, combination of chromosomal karyotype abnormality, patients who have used drugs harmful to the development of spermatozoa and patients with poor adherence | Semen analysis using WHO criteria - sperm concentration <5×10^6/ml, T<10.4 nmol/L, T/E2 ratio < 0.1 | Letrozole 2.5mg/day, tamoxifen 20mg/day | Placebo | 3 months | 3 months |

*FSH: Follicle-stimulating hormone , LH: Luteinizing hormone, T: Testosterone, PRL: Prolactin, P:Progesterone ,WHO: World Health Organisation, E: Estrogen, BMI: body mass index, HCT: haematocrit, SHBG: sex hormone binding globulin, INH-B

**Supplementary Table (2):** Risk of bias across individual randomised trials included in the network meta-analysis evaluating non-hormonal pharmacological treatment options for male infertility.

| Study | Randomisation | deviations from the intended interventions | Missing outcome data | Measurement of the outcome | Outcome selection | Blinding | Prospective registration | %loss to follow up |
| --- | --- | --- | --- | --- | --- | --- | --- | --- |
| Abel 1982 | High | Some concern | High | High | High | Not blinded | No | 43/196 |
| Boonyarangkul 2015 | High | Some concern | Some concern | High | High | Not blinded | No | 8/68 |
| Cavallini 2013 | Low | Low | Low | Low | Low | Double blinded | No | 4/52 |
| ElSheikh 2015 | High | Some concern | Some concern | High | High | Not blinded | No | 0 |
| Ghanem 2010 | Low | Low | Some concern | Some concern | High | Not blinded | No | 0 |
| Guo 2015 | Low | Some concern | Some concern | Low | High | Not blinded | No | 24/120 |
| Haje 2015 | High | Low | Low | High | High | Not blinded | No | 0 |
| Helo 2015 | Low | Low | Low | Low | Low | Double blinded | No | 2 |
| Krause 1992 | High | Some concern | Some concern | High | High | Not blinded | No | 0 |
| Mandal 2020 | High | High | High | Some concern | High | Single Blinded | No |  |
| Mičič 1985 | High | Some concern | Some concern | High | High | Not blinded | No | 0 |
| Moradi 2010 | Low | Low | Some concern | Some concern | High | Not blinded | No | 0 |
| Sokol 1988 | Some concern | Low | Some concern | Some concern | High | Not blinded | No | 0 |
| Xiaoxia 2019 | Some concern | Some concern | Low | High | Some concern | Not blinded | No | 0 |

**Supplementary Table (3):** Side-split analysis of inconsistency in the network for testosterone in the network meta-analysis evaluating non-hormonal pharmacological treatment options for male infertility.

| Intervention | Comparison | Direct | | Indirect |  | Difference | |  | tau |
| --- | --- | --- | --- | --- | --- | --- | --- | --- | --- |
|  |  | Coef. | Std. Err. | Coef. | Std. Err. | Coef. | Std. Err. | P value |  |
| Placebo | Tamoxifen | 0.99 | 0.34 | -0.73135 | 1.375 | 1.720425 | 1.416403 | 0.225 | 0.500021 |
| Placebo | Tamoxifen+Anti-oxidant | 2.02 | 0.54 | -0.57054 | 1.06 | 2.594858 | 1.217884 | 0.033* | 0.353333 |
| Anti-oxidant | Placebo | 0.06 | 0.51 | 2.654809 | 1.109 | -2.59486 | 1.217884 | 0.033* | 0.353333 |
| Anti-oxidant | Tamoxifen | 1.93 | 0.54 | -0.66579 | 1.061 | 2.594858 | 1.217884 | 0.033* | 0.353333 |
| Anti-oxidant | Tamoxifen+Anti-oxidant | 2 | 0.65 | -4.30787 | 12786 | 6.307744 | 12786.21 | 1 | 0.500015 |
| Aromatase-inhibitor | Placebo | -2.2 | 0.47 | -1.66518 | 0.744 | -0.55129 | 0.880093 | 0.531 | 0.589081 |
| Aromatase-inhibitor | Clomifene | 0.82 | 0.54 | -0.62908 | 0.458 | 1.445441 | 0.707517 | 0.041* | 0.348535 |
| Aromatase-inhibitor | Tamoxifen | -1.7 | 0.47 | -0.42744 | 0.574 | -1.29558 | 0.740737 | 0.08 | 0.413801 |
| Clomifene | Placebo | -1.7 | 0.33 | -3.17644 | 0.624 | 1.444754 | 0.707497 | 0.041* | 0.348534 |
| Tamoxifen | Tamoxifen+Anti-oxidant | 0.16 | 0.51 | 2.750058 | 1.112 | -2.59486 | 1.217883 | 0.033* | 0.353333 |

**Supplementary Table (4):** Summary of reported reproductive outcomes across included trials evaluating non-hormonal pharmacological treatment options for male infertility.

| Study | Intervention | n | Clinical Pregnancy | Comparison | n | Clinical Pregnancy |
| --- | --- | --- | --- | --- | --- | --- |
| Ghanem 2010 | Placebo | 30 | 4 | Clomifene+Supplement | 30 | 11 |
| Haje 2015 | Tamoxifen 20mg/d | 45 | 22 | L-Carnitine | 20 | 3 |
| Haje 2015 | Tamoxifen+L-Carnitine | 34 | 16 | Placebo | 29 | 6 |
| Mandal 2020 | Placebo | 100 | 4 | Clomifene | 100 | 22 |
| Sokol 1988 | Placebo | 9 | 4 | Clomifene | 11 | 1 |

**Supplementary Figure (1):** Summary of risk of bias across included randomised trials evaluating non-hormonal pharmacological treatment options for male infertility.

**Supplementary Figure (2):** Funnel plot evaluating the risk of publication bias in included randomised trials evaluating non-hormonal pharmacological treatment options for male infertility.

**
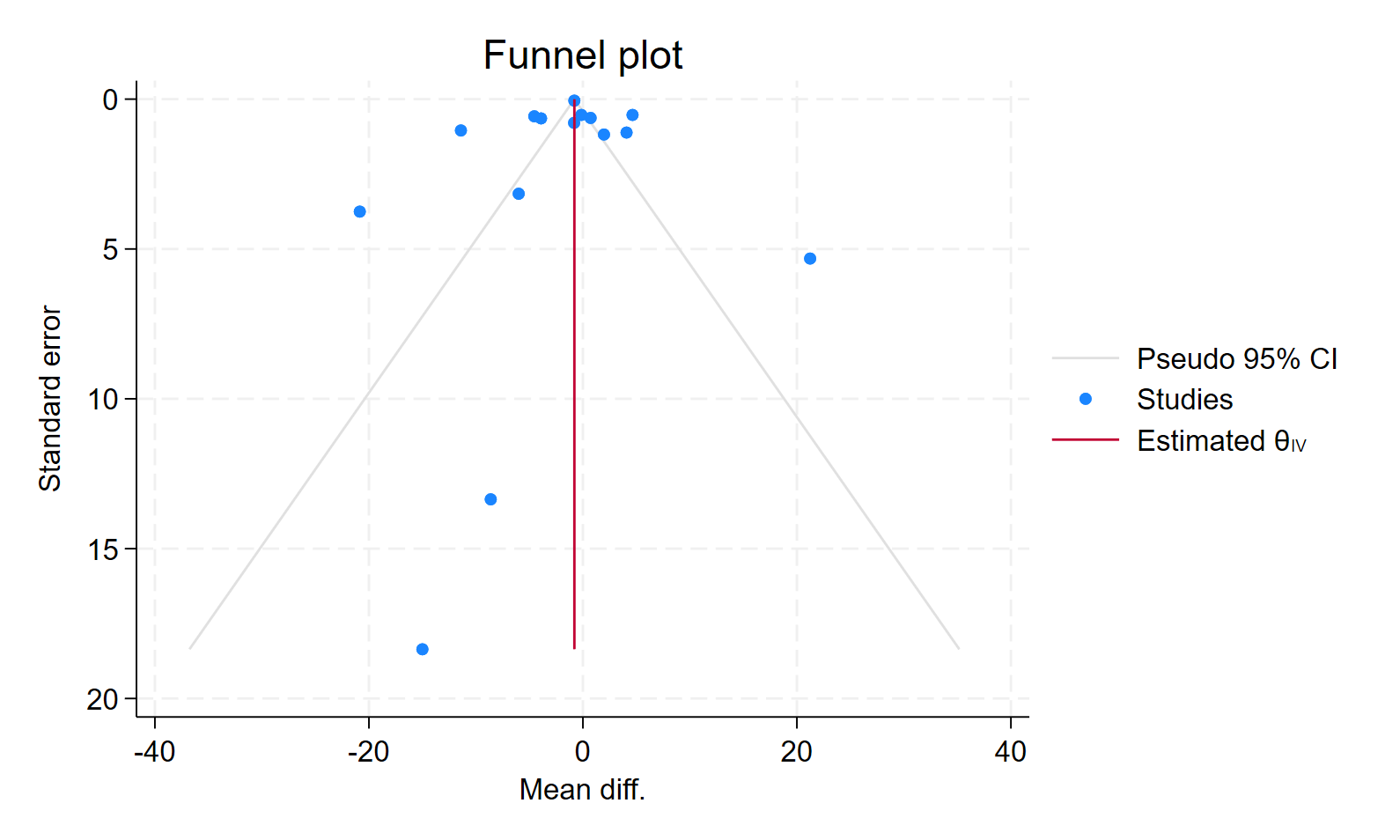
**

**Supplementary Figure (3):** Direct meta-analysis of changes in sperm concentration and motility with the use of Clomiphene vs placebo in men with infertility.

1. Sperm concentration

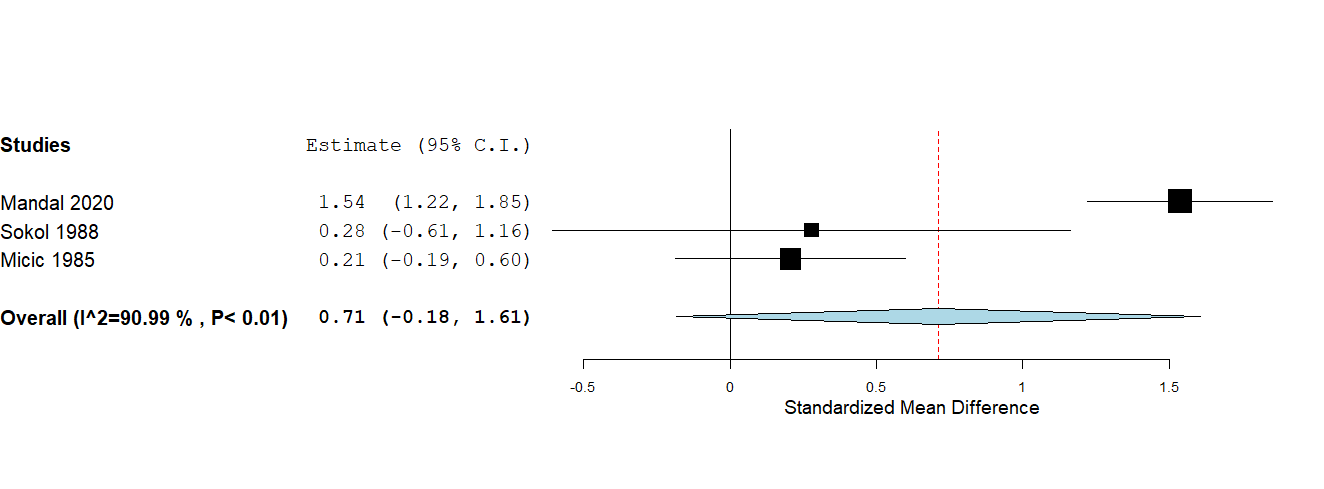

2. Sperm motility

**
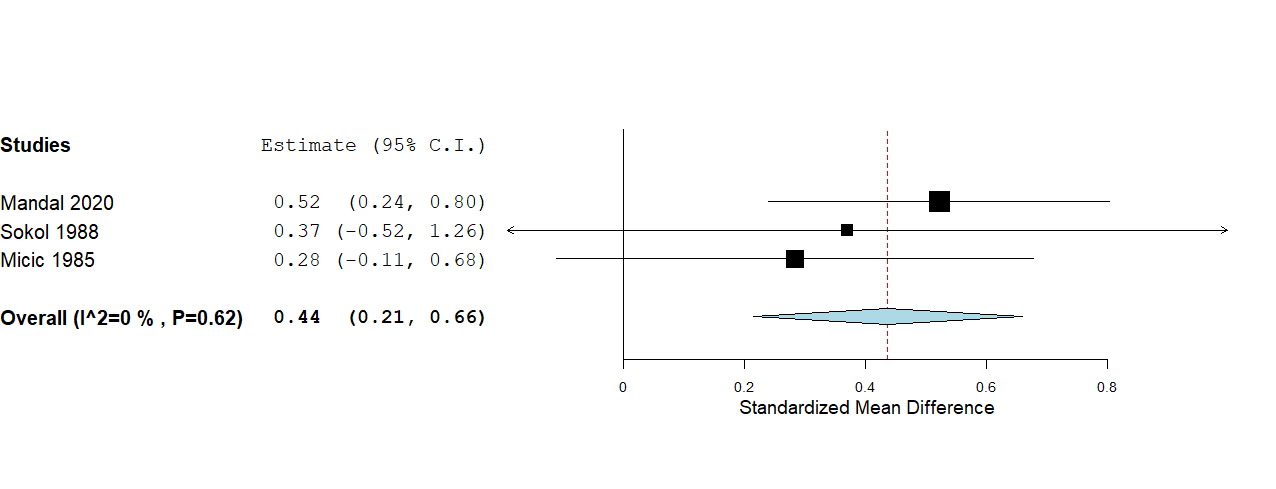
**

**Supplementary Figure (4):** Network meta-analysis of changes in sperm concentration across non-hormonal pharmacological treatment options for male infertility.

a-Network map

**
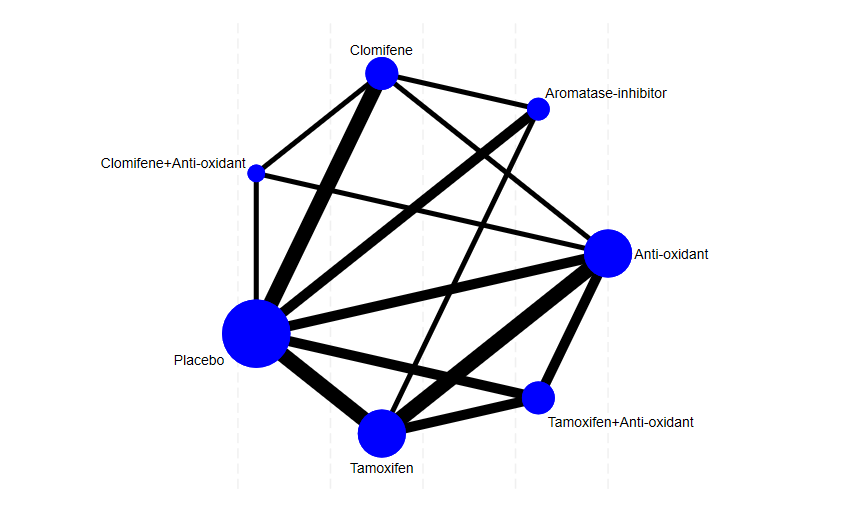
**

b-Forest Plot


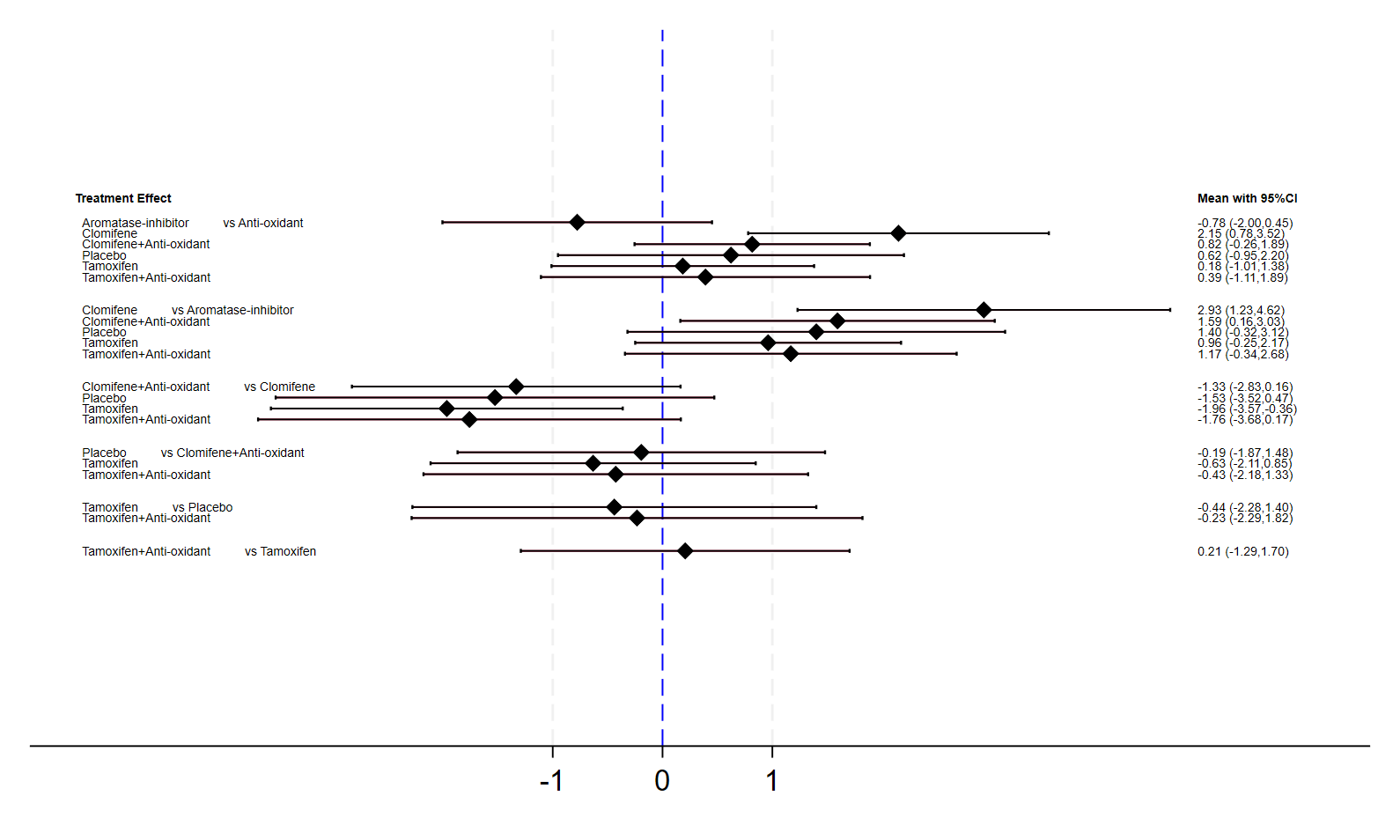


c-SCURA graph


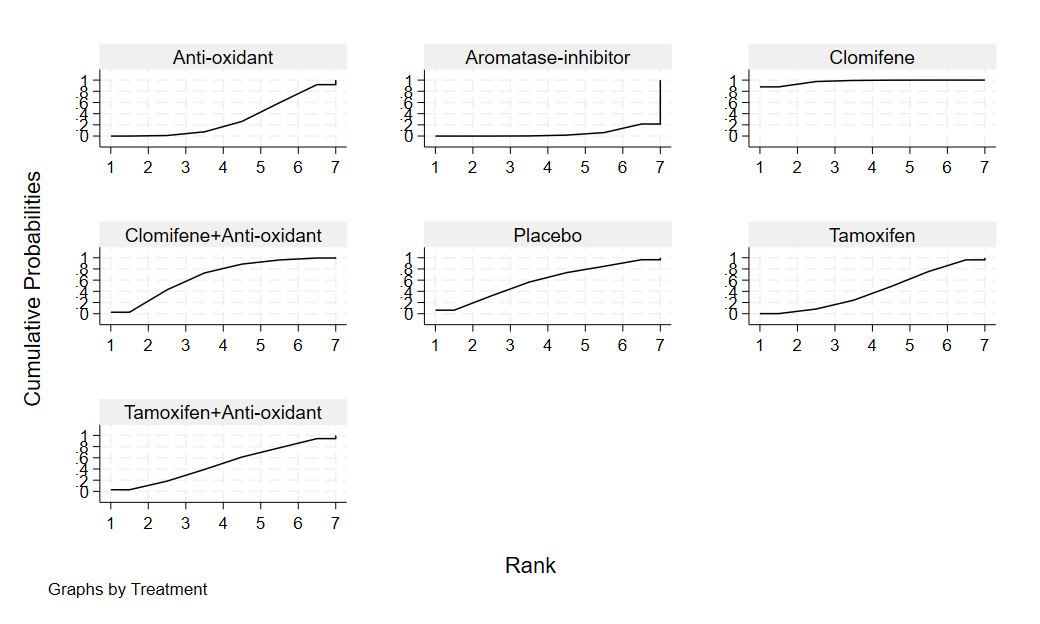


**Supplementary Figure (5):** Network meta-analysis of changes in sperm motility across non-hormonal pharmacological treatment options for male infertility.

a-Network map


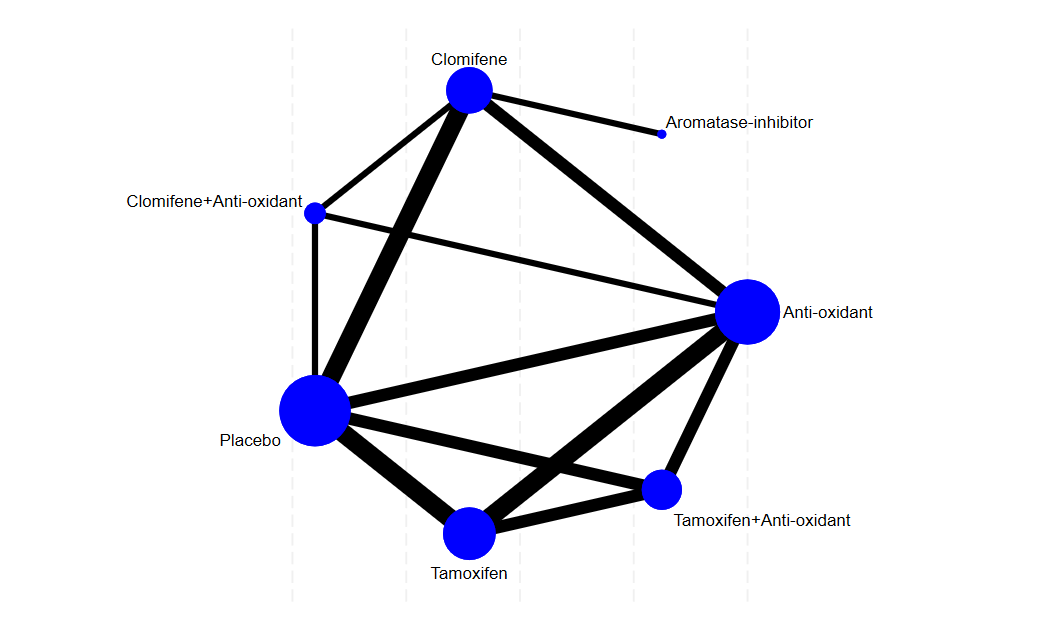


b-Forest Plot


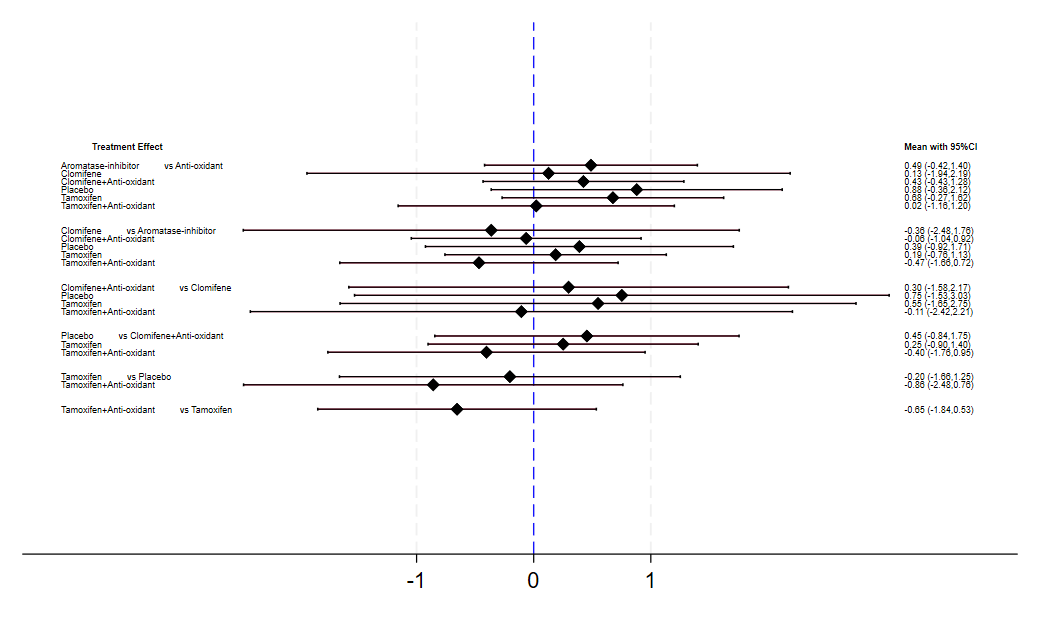


c-SCURA graph

**
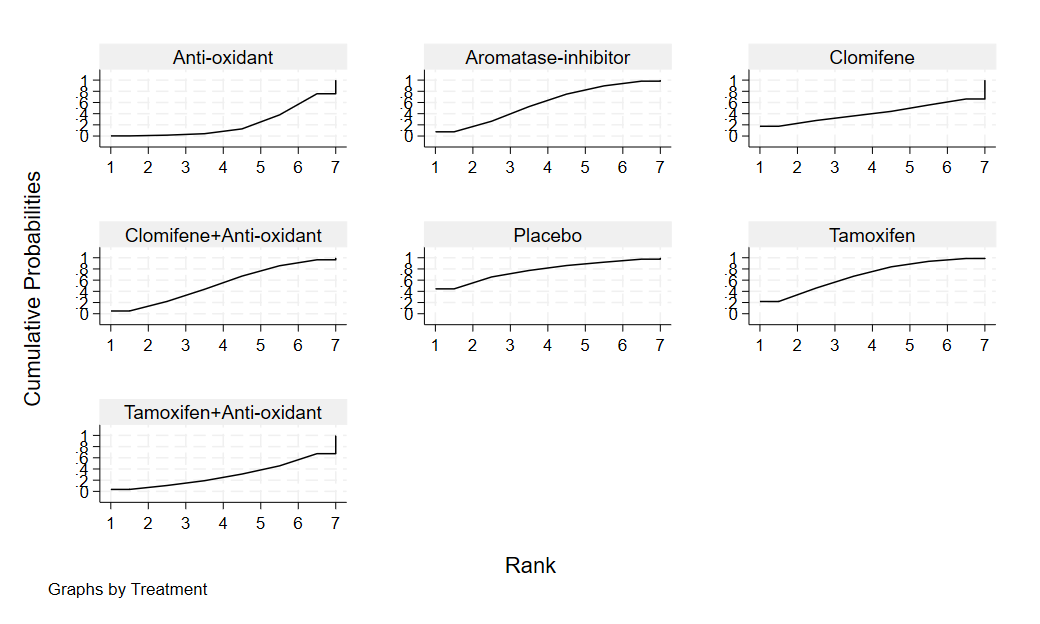
**

**Supplementary Figure (6):** Network meta-analysis of changes in semen volume across non-hormonal pharmacological treatment options for male infertility.

a-Network map


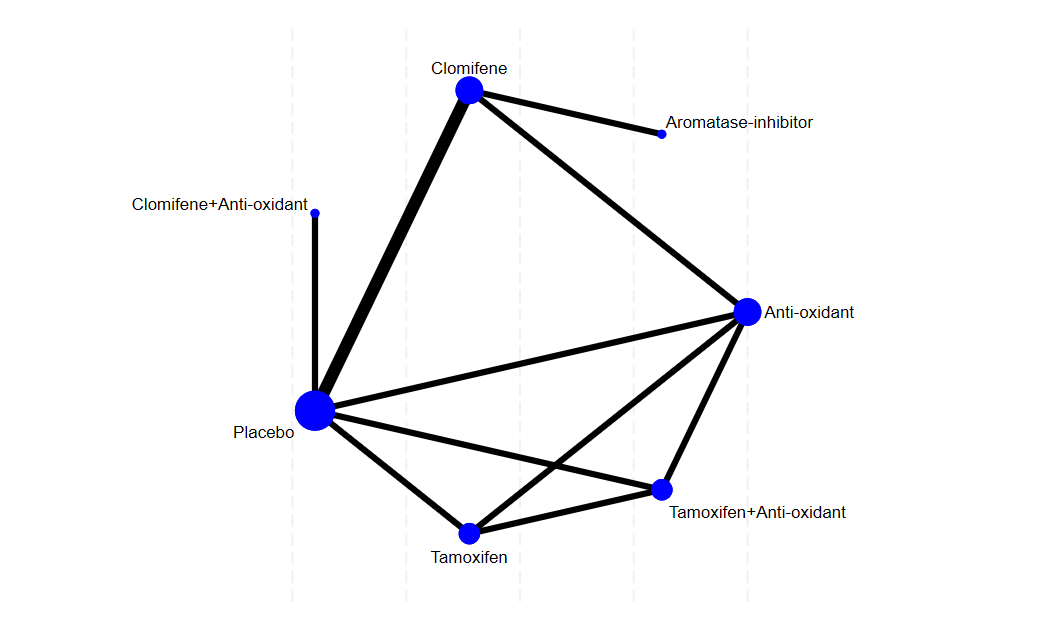


b-Forest Plot


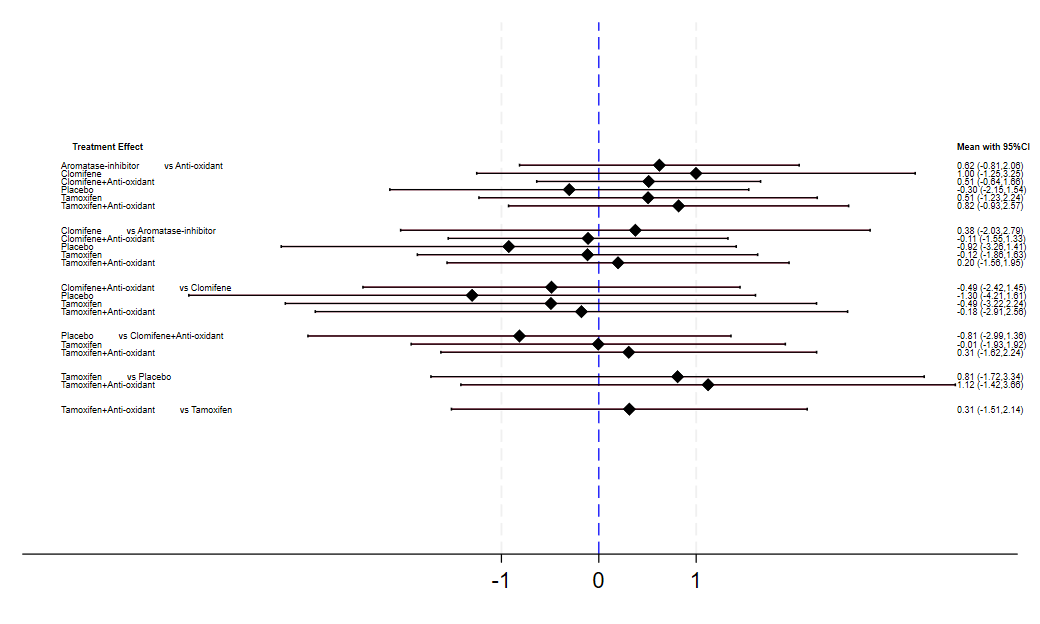


c-SCURA graph


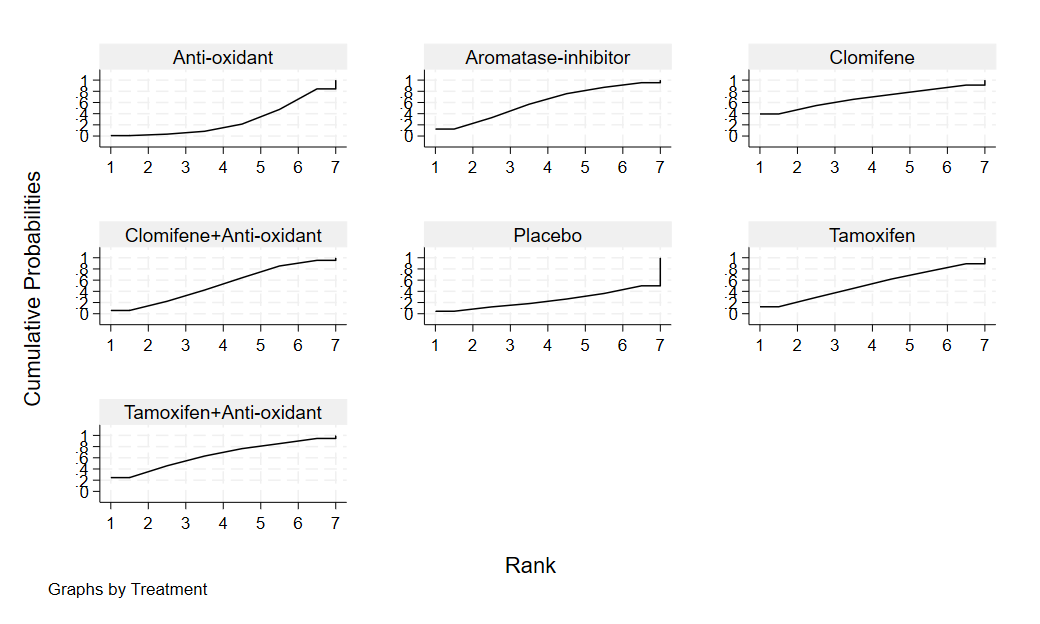


**Supplementary Figure (7):** Network meta-analysis of changes in normal sperm morphology across non-hormonal pharmacological treatment options for male infertility.

a-Network map


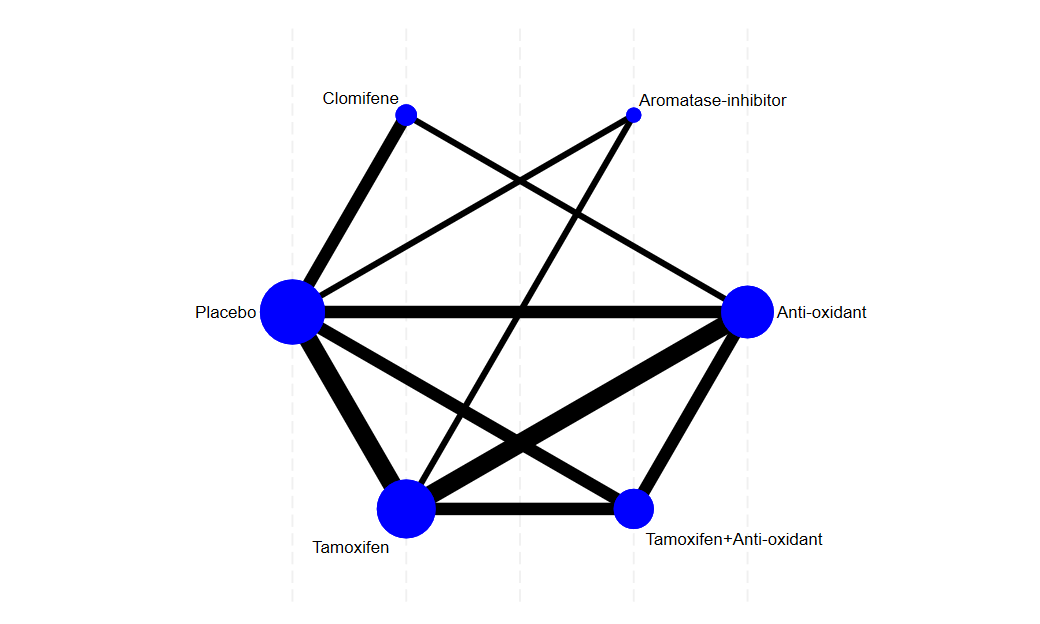


b-Forest Plot


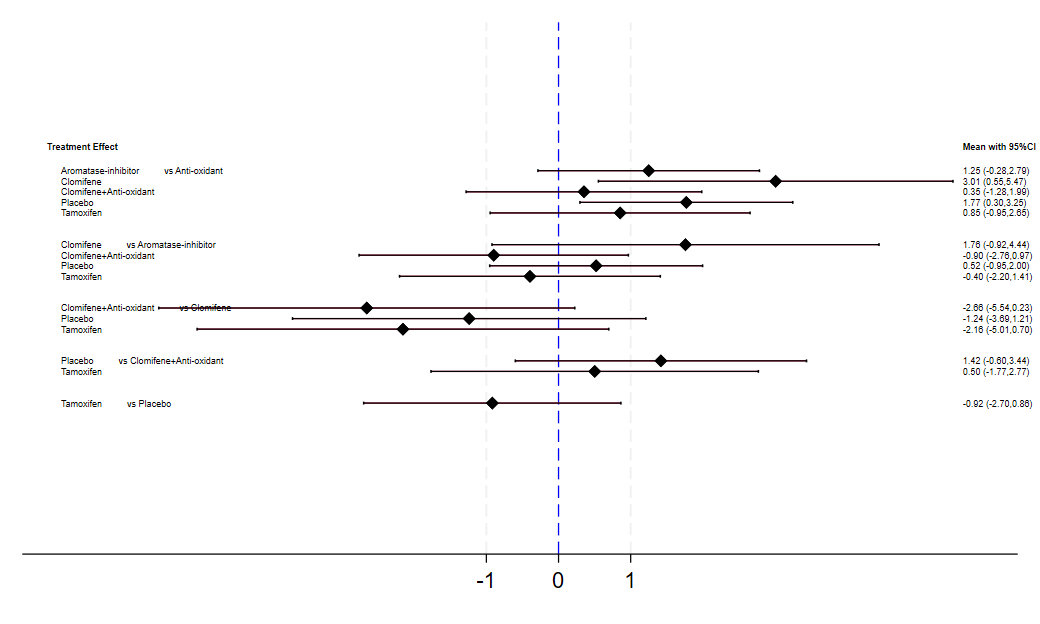


c-SCURA graph


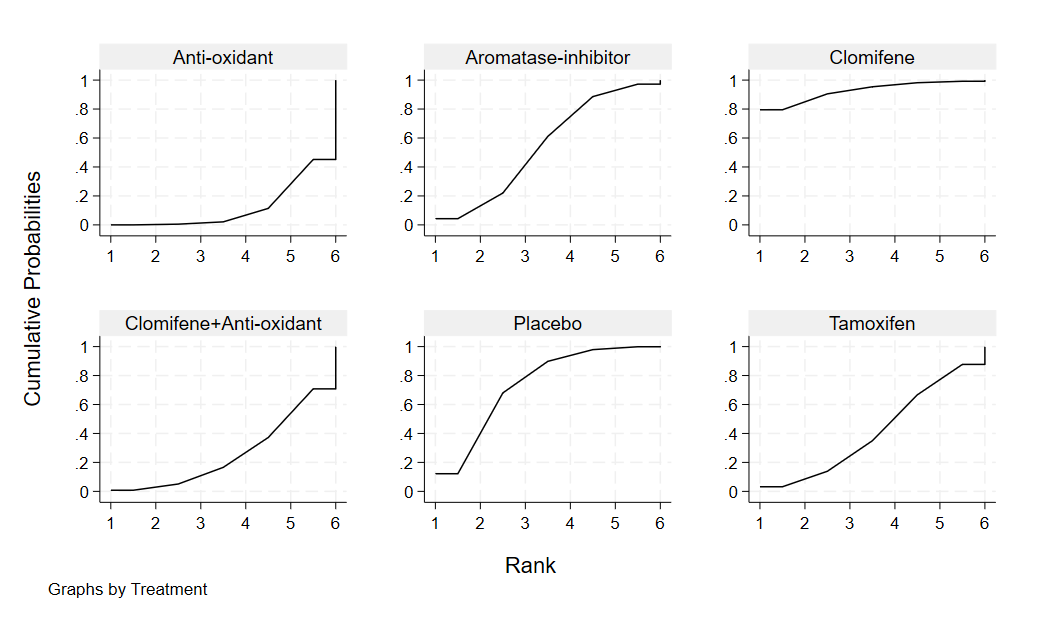


**Supplementary Figure (8):** Network meta-analysis of changes in FSH across non-hormonal pharmacological treatment options for male infertility.

a-Network map


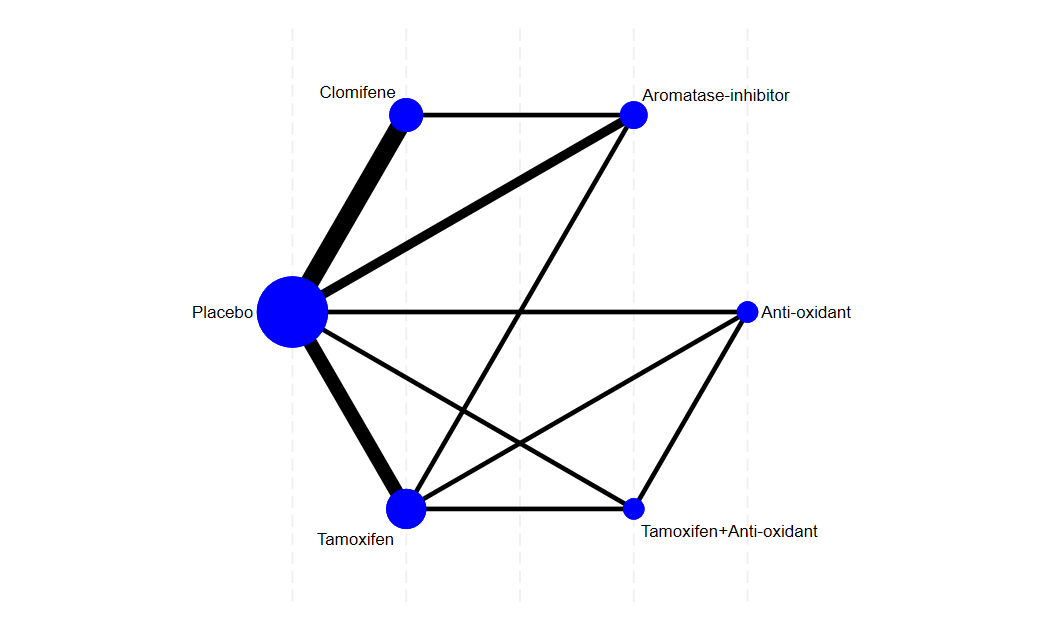


b-Forest Plot


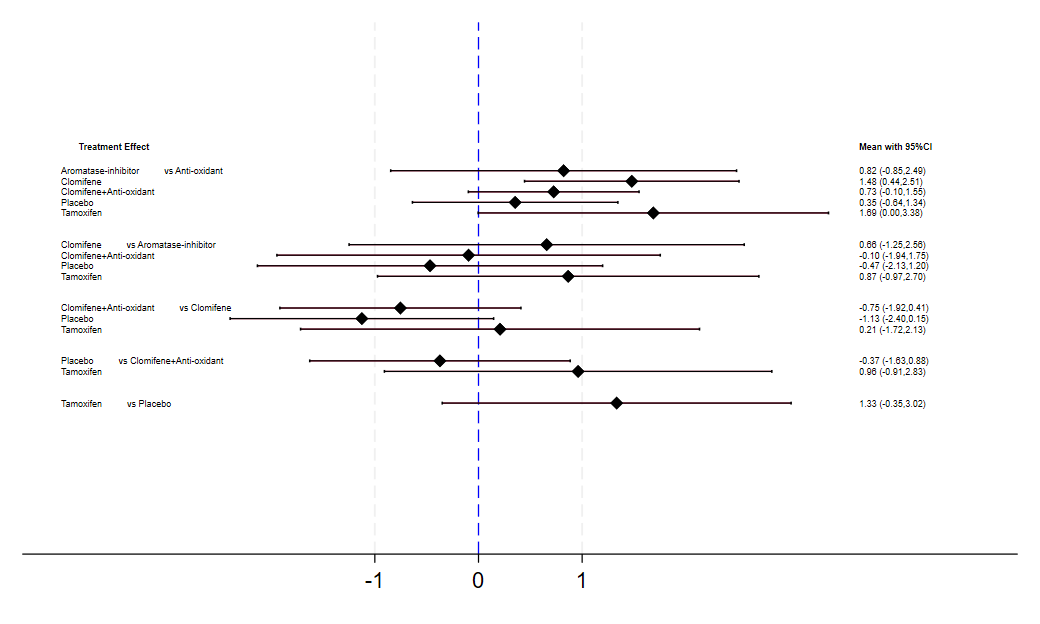


c-SCURA graph


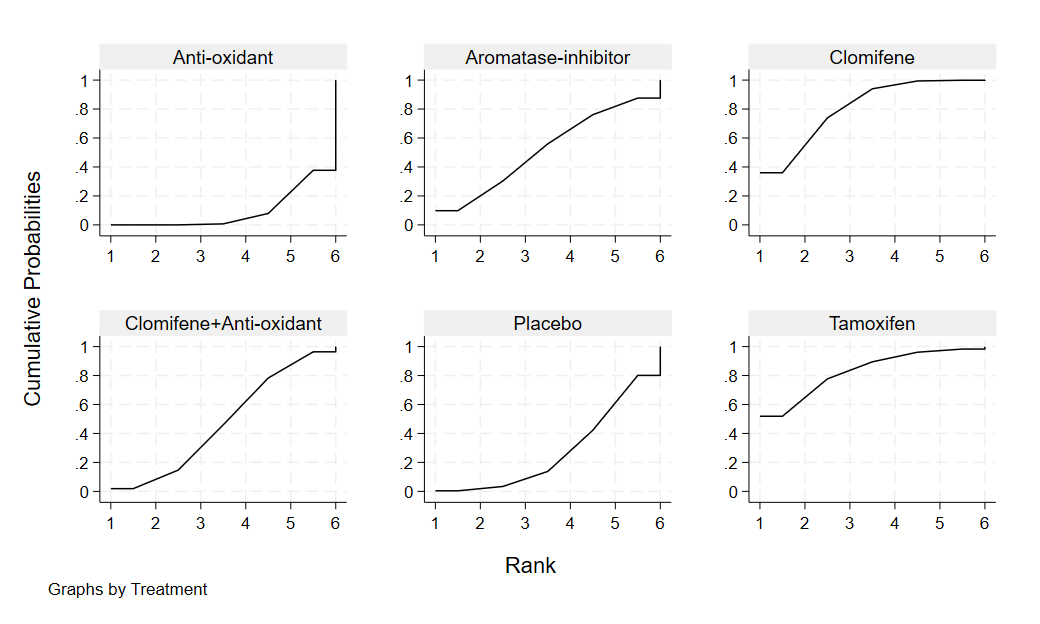


**Supplementary Figure (9):** Network meta-analysis of changes in LH across non-hormonal pharmacological treatment options for male infertility.

a-Network map


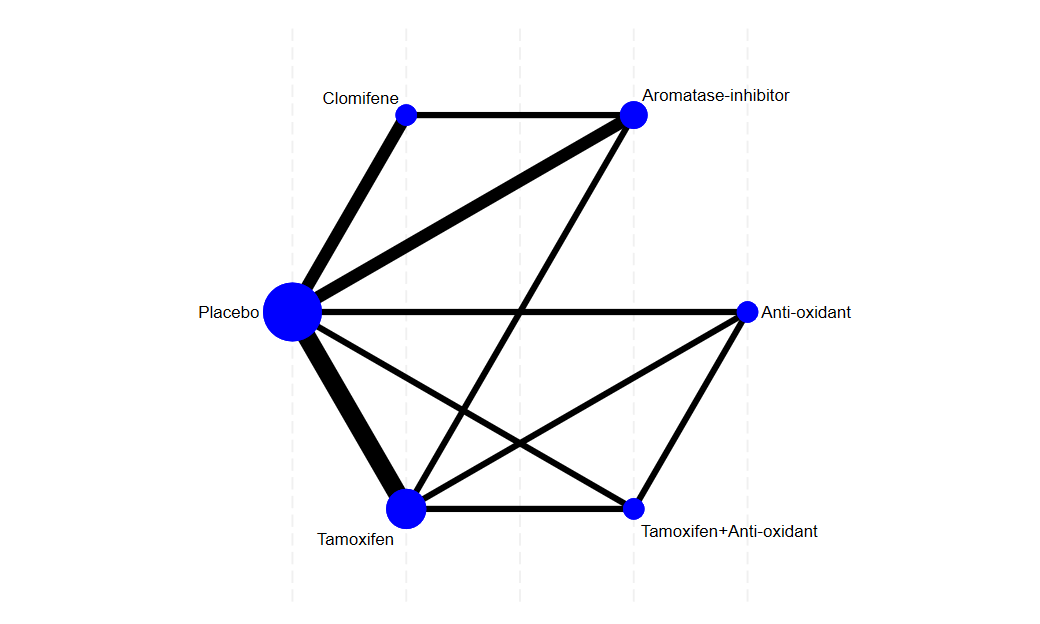


b-Forest Plot inconsistency model


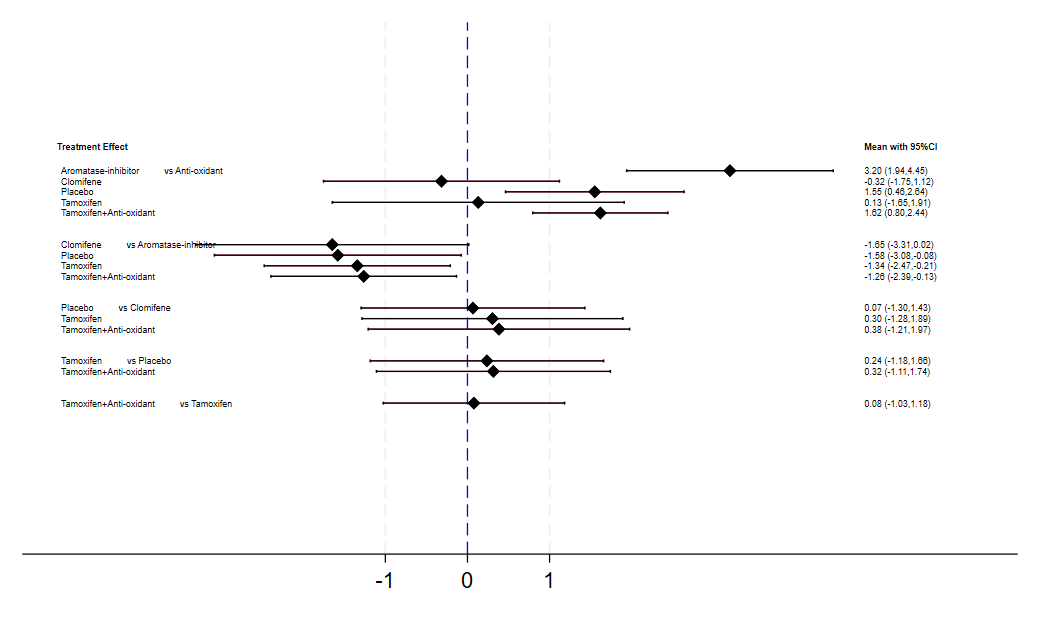


c-SCURA graph

**
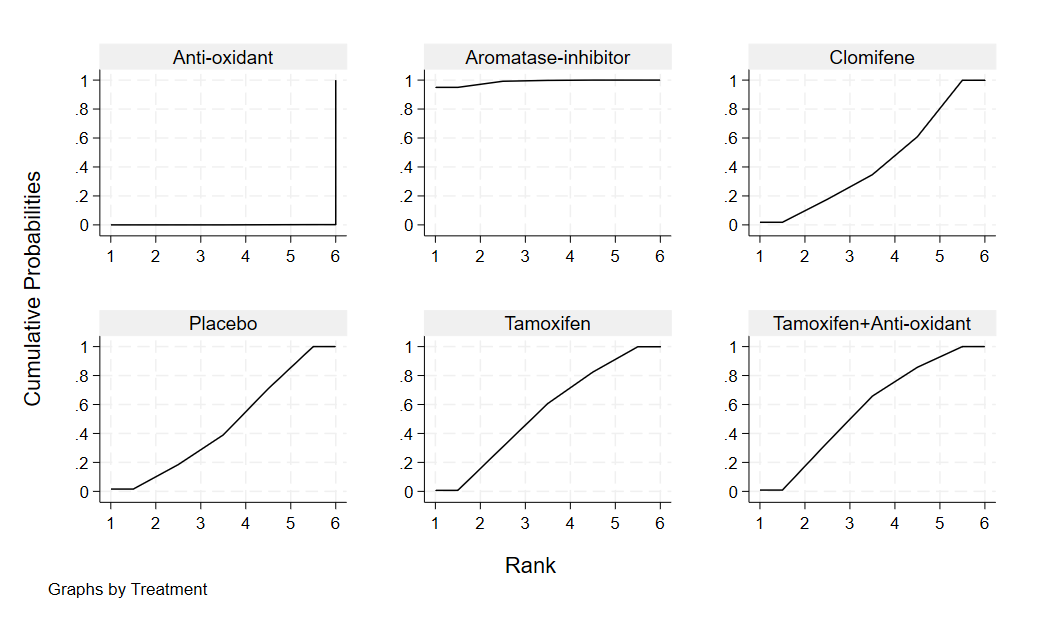
**

**Supplementary Figure (10):** Network meta-analysis of changes in testosterone across non-hormonal pharmacological treatment options for male infertility.

a-Network map


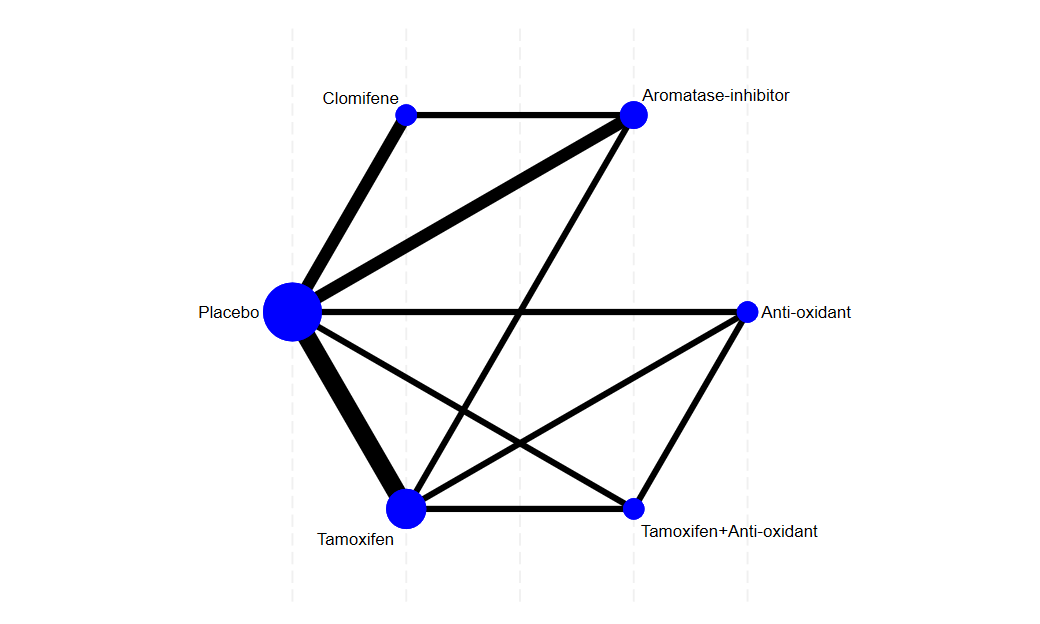


b-Forest Plot – inconsistency model


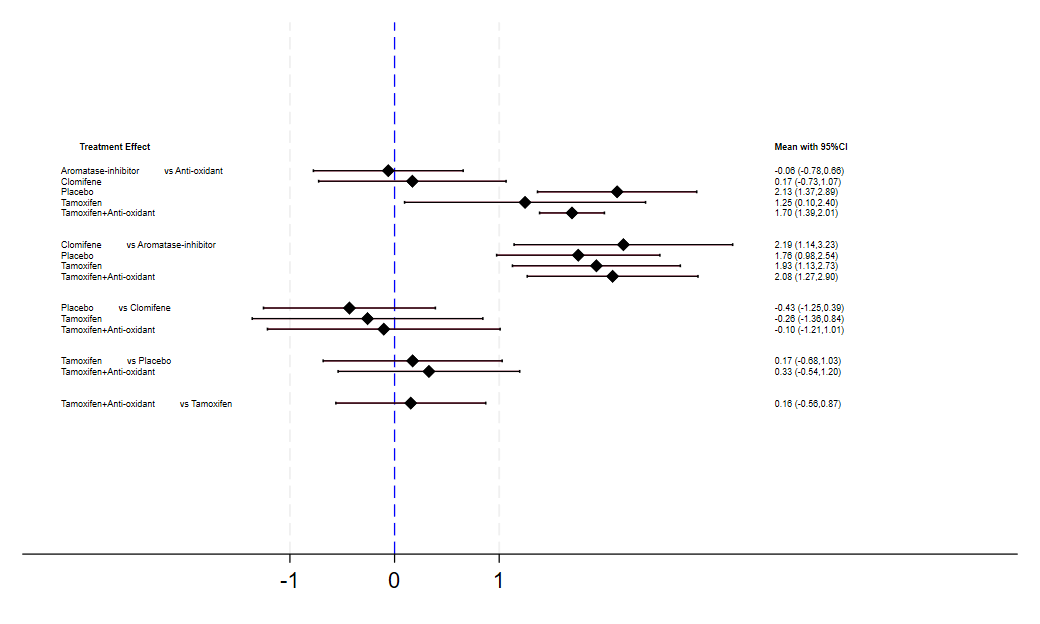


c-SCURA graph


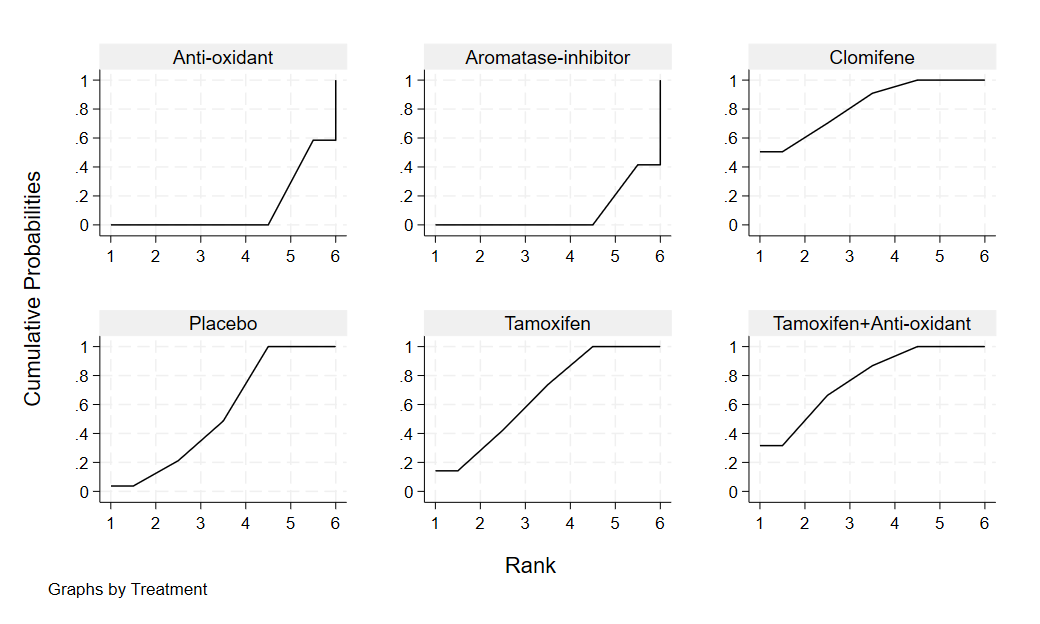

Supplement: Supplementary file 1 — Supplementary Material 1 [file 12894_2024_1545_MOESM1_ESM.docx]
